# Identification of an activation site in Bak and mitochondrial Bax triggered by antibodies

**DOI:** 10.1038/ncomms11734

**Published:** 2016-05-24

**Authors:** Sweta Iyer, Khatira Anwari, Amber E. Alsop, Wai Shan Yuen, David C. S. Huang, John Carroll, Nicholas A. Smith, Brian J. Smith, Grant Dewson, Ruth M. Kluck

**Affiliations:** 1 Molecular Genetics of Cancer Division, The Walter and Eliza Hall Institute of Medical Research, Victoria 3052, Australia; 2 Development and Stem Cells Program, Monash Biomedicine Discovery Institute and Department of Anatomy and Developmental Biology, Monash University, Melbourne, Victoria 3800, Australia; 3 Cancer and Haematology Division, The Walter and Eliza Hall Institute of Medical Research, Victoria 3052, Australia; 4 Department of Chemistry and Physics, La Trobe Institute for Molecular Sciences, La Trobe University, Victoria 3086, Australia; 5 Cell Signalling and Cell Death Division, The Walter and Eliza Hall Institute of Medical Research, Victoria 3052, Australia

## Abstract

During apoptosis, Bak and Bax are activated by BH3-only proteins binding to the α2–α5 hydrophobic groove; Bax is also activated via a rear pocket. Here we report that antibodies can directly activate Bak and mitochondrial Bax by binding to the α1–α2 loop. A monoclonal antibody (clone 7D10) binds close to α1 in non-activated Bak to induce conformational change, oligomerization, and cytochrome *c* release. Anti-FLAG antibodies also activate Bak containing a FLAG epitope close to α1. An antibody (clone 3C10) to the Bax α1–α2 loop activates mitochondrial Bax, but blocks translocation of cytosolic Bax. Tethers within Bak show that 7D10 binding directly extricates α1; a structural model of the 7D10 Fab bound to Bak reveals the formation of a cavity under α1. Our identification of the α1–α2 loop as an activation site in Bak paves the way to develop intrabodies or small molecules that directly and selectively regulate these proteins.

## Abstract (web summary)

During apoptosis, Bak and Bax are activated by BH3-only proteins binding to a specific hydrophobic groove. Here, the authors show that antibodies can also activate Bak and mitochondrial Bax by binding to the α1-α2 loop, thus identifying a potential clinical target.

## Graphical abstract

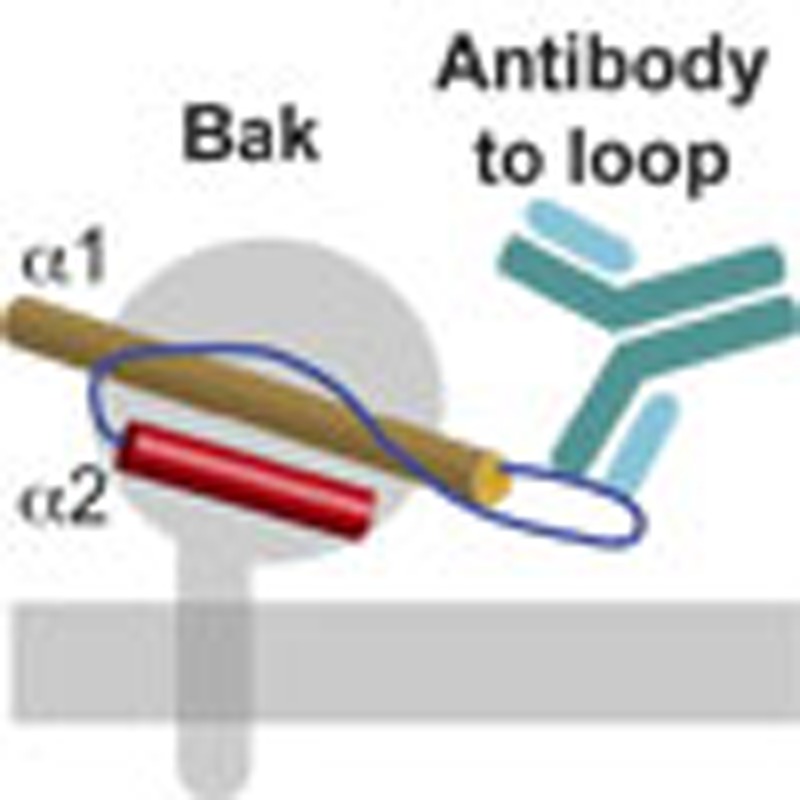

The commitment of cells to apoptotic cell death is determined by interactions between members of the Bcl-2 protein family on the mitochondrial outer membrane (MOM)^1^,^2^. Members of this family contain one to four Bcl-2 homology (BH) domains, and are divided into three sub-classes: prosurvival members that contain the BH1-BH4 domains; pro-apoptotic BH3-only members; and pro-apoptotic Bak and Bax that also contain the BH1-BH4 domains.

A key step in apoptosis is the loss of MOM integrity, which requires Bak and Bax activation followed by their structural conversion into pore-forming oligomers^2^,^3^,^4^. Both Bak and Bax contain nine α-helices, including a C-terminal transmembrane domain (α9), a buried BH3 domain (α2), as well as a hydrophobic surface groove (α2–α5) that can engage in interactions with other members of the Bcl-2 family. Whereas Bak is inherently mitochondrial, Bax is largely cytosolic with its α9-helix partly sequestered in the α2–α5 groove^5^ until Bax accumulates on the MOM following an apoptotic stimulus^6^,^7^.

Bak and Bax activation (that is, unfolding) are triggered when BH3-only proteins (for example, Bid or Bim) bind transiently to the α2–α5 groove^8^,^9^,^10^,^11^. In Bax, but not Bak, access of the activator to the α2–α5 groove requires initial binding to a second site (rear pocket) between α1 and α6 to displace α9 (refs 12, 13, 14). Bak may also be activated at sites other than the α2–α5 groove, as several proteins reported to directly activate Bak appear to lack a BH3-like motif^15^,^16^,^17^,^18^. Binding of BH3-only proteins to the Bak and Bax α2–α5 groove initiates unfolding of α2 followed by dissociation of both α1 and the α6–α8 latch^8^,^9^,^19^. The unfolded proteins collapse onto the mitochondrial surface and dimerize via a reciprocal BH3:groove interaction to nucleate the oligomers thought to permeabilize the MOM^5^,^20^,^21^,^22^,^23^,^24^,^25^.

Here we report the proximal α1–α2 loop as a second activation site in Bak and in mitochondrial Bax. This site can be targeted by antibodies to induce the same Bak and Bax homo-oligomerization and pore formation as that induced by BH3-only proteins. A structural model of the 7D10 Fab bound to Bak supports biochemical evidence that antibody binding to the α1–α2 loop acts by directly dissociating α1.

## Results

### An antibody to Bak triggers mitochondrial permeabilization

**Figure 1:**
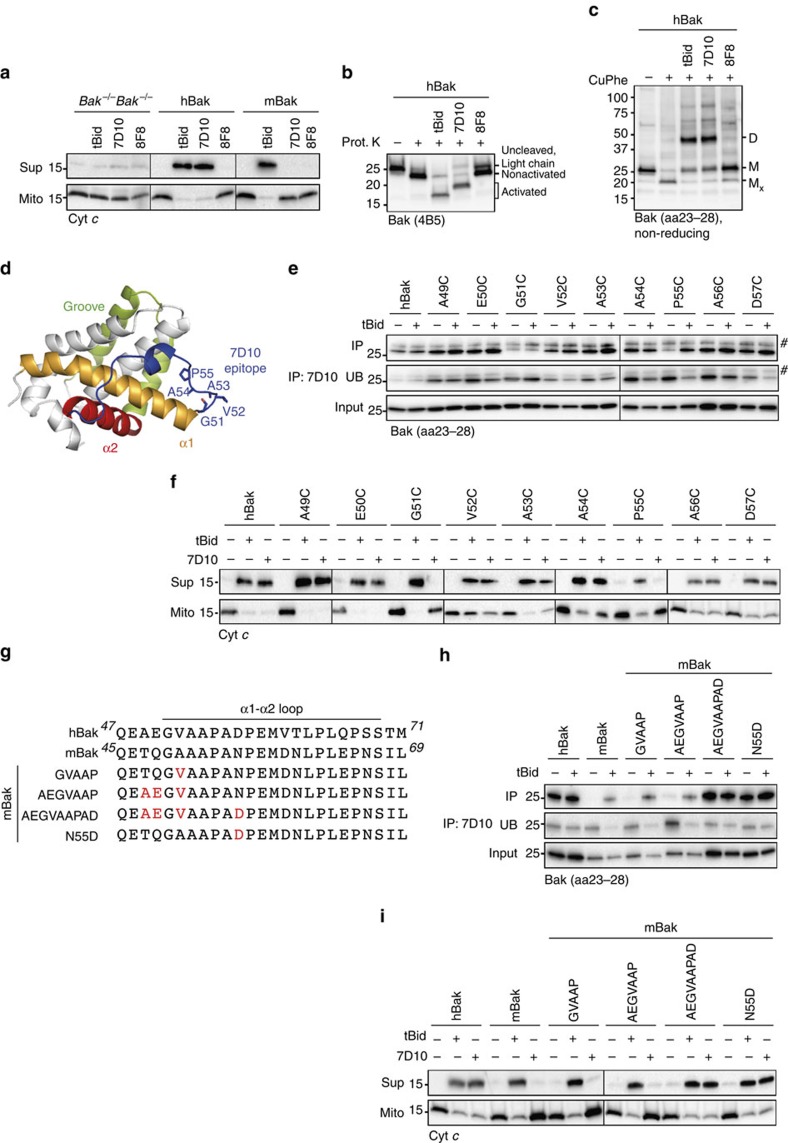
The 7D10 antibody triggers mitochondrial outer membrane permeabilization by binding to the α1–α2 loop of human Bak. (**a**) The 7D10 antibody induces cytochrome *c* release. Membrane fractions from *Bak*^*−/−*^*Bax*^*−/−*^ MEFs, those cells expressing human Bak (hBak), or *Bax*^*−/−*^ MEFs, were incubated with tBid or with the 7D10 or 8F8 antibodies. Supernatant (Sup) and pellet (Mito) fractions were assessed for cytochrome *c* release. (**b**) 7D10 triggers Bak conformational change as indicated by susceptibility to proteinase K. Incubations from **a** were treated with proteinase K and immunoblotted for Bak. Note that 7D10 binding at the loop masks a cleavage site (lane 4, Supplementary Fig. 1a), and that uncleaved Bak and light chain co-migrate. (**c**) 7D10 triggers Bak oligomerization. Incubations from **a** were treated with oxidant (CuPhe) to induce disulfide bond formation and immunoblotted for Bak. M, monomer; M_x,_ intramolecular linked monomers; D, intermolecular linked dimers. (**d**) The 7D10 trigger site in Bak is distinct from the canonical BH3-only trigger site. Cartoon representation of BakΔN19ΔC25 (2IMT, white) highlighting the α1–α2 loop (blue), and α3 and α4 of the hydrophobic groove (green). (**e**) Mutation of Bak G51 or P55 inhibits binding by 7D10. Membrane fractions from *Bak*^*−/−*^*Bax*^*−/−*^ MEFs expressing the indicated hBak variants were incubated with or without tBid followed by immunoprecipitation with 7D10 and immunoblotting for Bak. IP, immunoprecipitated; UB, unbound; #, light chain. (**f**) Mutation of Bak G51 or P55 prevent Bak activation and cytochrome *c* release by 7D10. Membrane fractions from **e** were incubated with tBid or 7D10 and assessed for cytochrome *c* release. (**g**) Substitutions in mouse Bak to generate the 7D10 epitope. (**h**) The ^51^GVAAPAD^57^ sequence allows binding of 7D10 to mouse Bak. Membrane fractions from *Bax*^*−/−*^ MEFs (mBak cells), or *Bak*^*−/−*^*Bax*^*−/−*^ MEFs expressing hBak or the indicated mBak variants, were incubated with or without tBid followed by immunoprecipitation with 7D10 and immunoblotting for Bak. (**i**) The ^51^GVAAPAD^57^ sequence allows 7D10 to activate mouse Bak and release cytochrome *c*. Membrane fractions from **h** were incubated with tBid or 7D10 and assessed for cytochrome *c* release. In **a**–**c**,**e**,**f**,**h** and **i** data are representative of three independent experiments.

While using antibodies to characterize Bak conformational changes triggered by tBid, we found that an anti-Bak antibody, clone 7D10, could trigger cytochrome *c* release from mitochondria expressing human Bak (hBak, Fig. 1a). During the incubation, Bak had become activated as shown by sensitivity to limited proteolysis (Fig. 1b; Supplementary Fig. 1a–c), and had oligomerized as shown by disulfide-linked dimers induced by addition of the oxidant copper phenanthroline (CuPhe, Fig. 1c). Two alternative antibodies, 8F8 and anti-FLAG, that bound Bak N-terminal to α1, failed to activate Bak and FLAG-Bak, respectively (Fig. 1a–c; Supplementary Fig. 2a,b). These data demonstrate that an antibody can trigger Bak activation, oligomerization and mitochondrial cytochrome *c* release, and that the epitope recognized by 7D10 may be an important site for activating Bak.

### The 7D10 antibody binds to the α1–α2 loop of human Bak

7D10 is a rat monoclonal antibody raised against human BakΔC25 (ref. 22). By peptide array we had defined ^51^GVAAP^55^ at the start of the α1–α2 loop (Fig. 1d) as the minimal set of residues required for 7D10 binding, with G51 and P55 as particularly important residues within this sequence^19^. We then tested whether substituting each residue in this region (with cysteine) decreased 7D10 binding and activation. When tested by immunoprecipitation, G51 and P55 were confirmed to be important for 7D10 binding (Fig. 1e) and for 7D10-induced cytochrome *c* release (Fig. 1f). Curiously, 7D10 could immunoprecipitate BakP55C only after its activation by tBid (Fig. 1e), suggesting that cysteine at position 55 is permissive for binding of 7D10, but that proline at that position helps to orientate the epitope in non-activated Bak. The substitutions did not affect tBid-induced cytochrome *c* release (Fig. 1f), or Bak-mediated apoptosis in cells (Supplementary Fig. 3a), although we cannot exclude the possibility that variants may have slightly more or less function than wild-type Bak.

To test if ^51^GVAAP^55^ was sufficient for Bak activation by 7D10, four sets of substitutions were made in mouse Bak (mBak, Fig. 1g), which we had found was not activated by 7D10 (Fig. 1a). The substitutions did not affect Bak-mediated apoptosis in cells (Supplementary Fig. 3b). In mitochondria experiments, the GVAAP and AEGVAAP sequences were not sufficient for 7D10 to immunoprecipitate non-activated mBak or induce cytochrome *c* release, whereas the GAAAPAD sequence was sufficient (Fig. 1h,i). The importance of the aspartate residue was confirmed by the single substitution (N55D) allowing mBak binding and activation by 7D10 (Fig. 1h,i), and by the reverse substitution (D57N) in hBak inhibiting activation by 7D10 (Supplementary Fig. 4a–c). Thus, as discussed for the proline residue, this aspartate residue may help ‘present' other epitope residues in non-activated hBak. In addition, both the hBak P55C and D57C variants were susceptible to proteinase K even before their activation (Supplementary Fig. 5a), suggesting that P55 and D57 structure the α1–α2 loop in a way that allows recognition by 7D10 but not proteinase K. Further, D57 is not in contact with the antibody, as shown by the ability of D57C to disulfide bond (D57C:D57C linkage) between Bak molecules after 7D10 has bound and triggered Bak activation (Supplementary Fig. 5b,c). Similarly, the A49C:A49C linkage after 7D10 activation indicates that the other end of the epitope is delimited by A49 (Supplementary Fig. 5b,c). Thus, the functional 7D10 epitope in non-activated Bak is the ^51^GVAAPAD^57^ sequence, with P55 and D57 orientating other epitope residues (for example, G51) that directly contact the antibody.

### Antibody-triggered activation requires epitope close to α1

**Figure 2:**
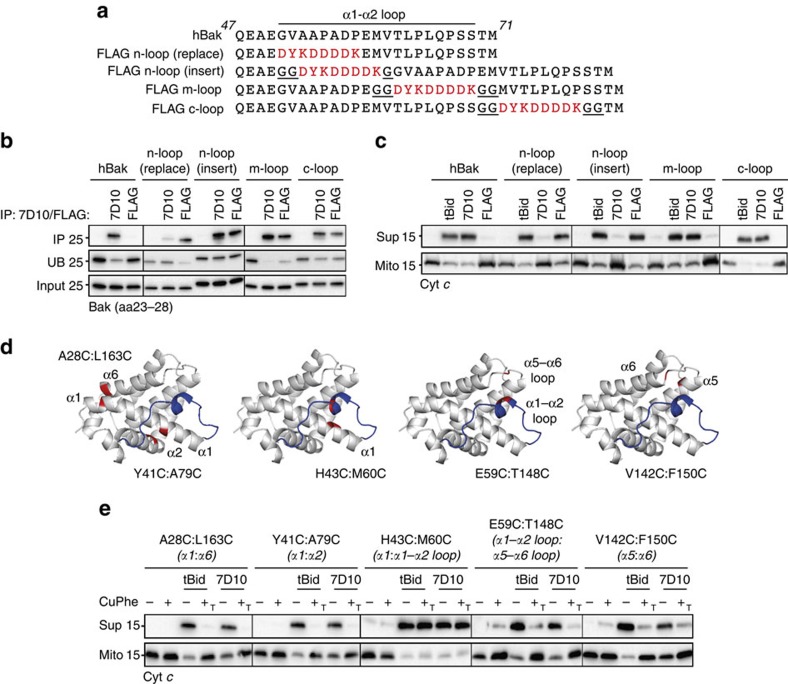
Antibody-triggered Bak activation requires proximity of the epitope to α1. (**a**) The FLAG epitope at four positions in the Bak α1–α2 loop. The FLAG epitope (red) and glycine residues (underlined) added to optimize epitope presentation are highlighted. (**b**) The FLAG epitopes allow recognition by anti-FLAG antibody. Membrane fractions from *Bak*^*−/−*^*Bax*^*−/−*^ MEFs expressing the indicated hBak variants were immunoprecipitated with the 7D10 or anti-FLAG antibodies and immunoblotted for Bak. IP, immunoprecipitated; UB, unbound. (**c**) Only epitopes positioned next to α1 allow cytochrome *c* release by related antibodies. Membrane fractions from **b** were incubated with tBid, 7D10 or anti-FLAG antibody and assessed for cytochrome *c* release. (**d**) Disulfide tethers introduced at five positions in Bak. Cartoon representation of BakΔN19ΔC25 (2IMT, white) highlighting the α1–α2 loop (blue) and cysteine substitutions (red) for each disulfide tether. (**e**) A tether between the 7D10 epitope and α2 indicates 7D10 does not act directly on α2. Membrane fractions from *Bak*^*−/−*^*Bax*^*−/−*^ MEFs expressing the indicated double cysteine variants were pre-incubated with CuPhe to induce a tether (T; Supplementary Fig. 6a), and then incubated with tBid or 7D10 and assessed for cytochrome *c* release. Note that the H43C:M60C tether lies between the 7D10 epitope and α2, but does not block 7D10 activation of Bak. In **b**,**c** and **e** data are representative of three independent experiments.

To investigate the mechanism of antibody-triggered Bak activation and in particular the relevance of the epitope being close to α1, the FLAG epitope was placed in four positions in the α1–α2 loop (Fig. 2a). Each variant retained pro-apoptotic function in cells (Supplementary Fig. 3c), and each variant could be immunoprecipitated with anti-FLAG antibody (Fig. 2b). Notably, antibody-triggered cytochrome *c* release occurred only if the FLAG epitope was N-terminal in the α1–α2 loop (n-loop replacement or insertion; Fig. 2c). Moreover, when the 7D10 epitope was re-positioned 11 residues away from α1, 7D10 could bind but could no longer activate Bak (n-loop insert, Fig. 2b,c). As α1 dissociation is an important step in Bak activation by tBid^19^, the anti-FLAG and 7D10 antibodies may act by binding close to α1 and directly destabilizing its many contacts with the remainder of Bak.

### 7D10 binding to Bak does not directly target α2

To test which Bak conformational changes mediated 7D10-induced activation of Bak, we used disulfide tethers to restrain the various structural elements in Bak (Fig. 2d). Each tether was efficient and did not hinder 7D10 binding to Bak (Supplementary Fig. 6a,b). Three tethers (A28C:L163C, Y41C:A79C and V142C:T148C) previously shown to block Bak activation by tBid^8^,^19^,^23^ also blocked activation initiated by 7D10 (Fig. 2e), indicating that although tBid and 7D10 bind to different sites on Bak (Fig. 1d), most of the subsequent unfolding events (illustrated in Supplementary Fig. 6c) are equivalent. A tether of the α1–α2 loop to the α5–α6 loop (E59C:T148C), similar to a tether (L45C:M137C) shown to block Bax activation^12^, also blocked Bak activation by both tBid and 7D10 (Fig. 2e). This tether may prevent core/latch separation, although the tether is to the hinge region rather than to the latch (α6–α8) itself. In contrast, because the α1–α2 loop lies across α1, tethering the loop would greatly hinder α1 dissociation. The final tether, from the middle of the α1–α2 loop to α1, was designed to prevent the effect of antibody binding to the N-terminal end of the loop from being transduced to the C-terminal end of the loop and to α2 (H43C:M60C; Fig. 2d). In spite of this tether, 7D10 still activated Bak (Fig. 2e), further indicating that 7D10 binding directly impacts α1, and not α2.

### The 7D10 Fab efficiently activates Bak

To test whether the bivalency or bulk (∼150 kDa) of the 7D10 antibody might contribute to Bak activation, the 7D10 antibody fragment (Fab, ∼50 kDa) was purified (Supplementary Fig. 7a,b). When Fab was incubated with mitochondria, Bak underwent activation and oligomerization (Supplementary Fig. 7c) and released cytochrome *c* with similar molar efficiency as the full antibody (Supplementary Fig. 7d). Thus, the bivalency and bulk of 7D10 are not essential for its activation of Bak.

### The 7D10 Fab alters oocyte mitochondrial morphology

The 7D10 Fab was then tested in cells by microinjection into immature human oocytes. The 7D10 Fab, but not a control Fab, induced mitochondrial aggregation that progressed over 16 h (Supplementary Fig. 8a–c). The morphology was consistent with mitochondrial fusion, supported by a decrease in mitochondrial particle number and increase in size (Supplementary Fig. 8f,g). We also observed an apparent deformation of the plasma membrane creating a highly contoured surface compared with controls (Supplementary Fig. 8d,e). This is consistent with early stages of apoptosis seen in oocytes under other conditions^26^. Importantly, changes in mitochondrial morphology were not apparent when the 7D10 Fab was injected into mouse oocytes (Supplementary Fig. 9a–e), consistent with an on target effect of the Fab on human Bak. Notably, the human oocytes appear not to have undergone MOMP, as TMRM was retained in the aggregated mitochondria (Supplementary Fig. 8a–c). Further, mitochondrial fission rather than fusion most often accompanies MOMP^27^,^28^, although Bak and Bax may also contribute to mitochondrial fusion^29^,^30^,^31^.

### Mitochondrial Bax can be activated by antibody to α1–α2 loop

**Figure 3:**
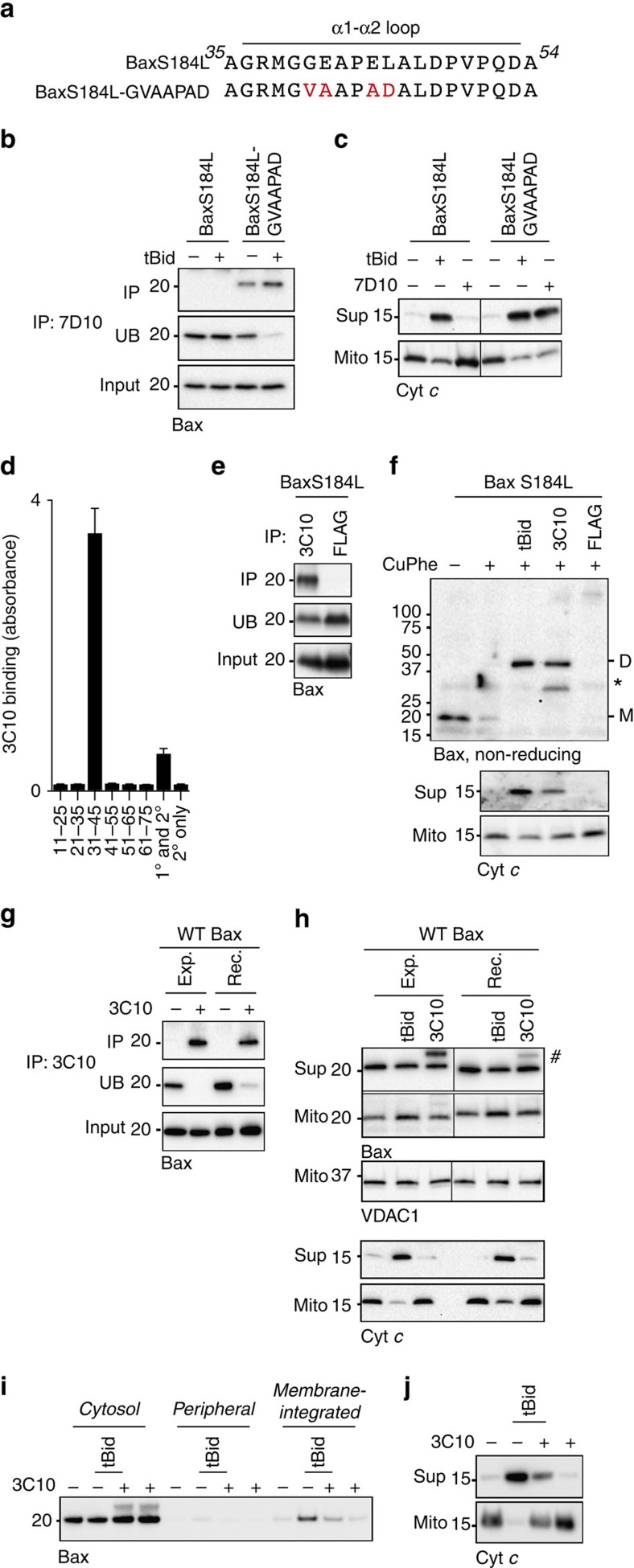
Bax at mitochondria can also be activated by an antibody to the α1–α2 loop. (**a**) Substitutions in mitochondria-targeted Bax to generate the 7D10 epitope. The α1–α2 loop in BaxS184L highlighting substitutions (red). (**b**,**c**) The 7D10 epitope allows 7D10 to bind BaxS184L and trigger cytochrome *c* release. Membrane fractions from *Bak*^*−/−*^*Bax*^*−/−*^ MEFs expressing either of the BaxS184L variants were incubated with tBid or 7D10, and tested for 7D10 immunoprecipitation of Bax (**b**) and for cytochrome *c* release (**c**). (**d**) The 3C10 epitope maps to the Bax α1–α2 loop, close to α1. Immunoreactivity of the rat monoclonal antibody, clone 3C10, towards biotinylated human Bax peptides as determined by enzyme-linked immunosorbent assay. *X* axis indicates Bax residue number or control reactions. Mean and s.d. of three independent experiments. (**e**) 3C10 binds non-activated BaxS184L. Membrane fractions from *Bak*^*−/−*^*Bax*^*−/−*^ MEFs expressing BaxS184L were immunoprecipitated with the 3C10 or anti-FLAG (negative control) antibodies and immunoblotted for Bax. (**f**) 3C10 activates and oligomerizes BaxS184L to release cytochrome *c*. Membrane fractions from **e** were incubated with tBid, 3C10 or anti-FLAG and assessed for disulphide-linked dimers (D, upper panel) and for cytochrome *c* release (lower panel). Note that after CuPhe addition, the monomer (M) appears to link to very high-order species. (*, non-specific band) (**g**) 3C10 binds WT Bax. Lysates of *Bak*^*−/−*^*Bax*^*−/−*^ MEFs expressing Bax (Exp.) or of *Bak*^*−/−*^*Bax*^*−/−*^ membranes reconstituted with recombinant Bax (100 nM, Rec.) were immunoprecipitated with 3C10 and immunoblotted for Bax. (**h**) 3C10 fails to trigger WT Bax translocation to mitochondria and release of cytochrome *c*. Permeabilized *Bak*^*−/−*^*Bax*^*−/−*^ MEFs expressing hBax (Exp.) or *Bak*^*−/−*^*Bax*^*−/−*^ MEF membranes reconstituted with recombinant Bax (50 nM, Rec.) were incubated with tBid or 3C10. Supernatant (Sup) and pellet (Mito) fractions were immunoblotted for Bax, VDAC or cytochrome *c*. (#, light chain). (**i**,**j**) 3C10 blocks Bax translocation and integration and cytochrome *c* release triggered by tBid. *Bak*^*−/−*^ mouse liver mitochondria were incubated as in **h**, but an additional sample also contained both 3C10 and tBid. Samples were assessed for Bax membrane integration (**i**), or for cytochrome *c* release (**j**) as in (**h**). In **b**,**c** and **e**–**j** data are representative of three independent experiments.

Due to the structural and functional similarities of Bak and Bax, we anticipated that Bax would also be activated by an antibody to its α1–α2 loop. This was first tested in a mitochondrial form of Bax (S184L), that like Bak, adopts a non-activated conformation in which α9 forms a transmembrane domain^32^,^33^,^34^, and that converts to the same activated conformation as Bax in response to tBid^25^. Because of its mitochondria location, BaxS184L does not require initial binding of tBid to the rear pocket to extrude α9 (refs 12, 13, 14). When the 7D10 epitope was positioned in the α1–α2 loop of mitochondrial Bax (BaxS184L-GVAAPAD; Fig. 3a), the 7D10 (anti-Bak) antibody could bind (Fig. 3b) and release cytochrome *c* (Fig. 3c). In addition, a novel anti-Bax antibody, clone 3C10, that recognized a peptide encompassing residues 31–45 at the start of Bax α1–α2 loop (Fig. 3d) was able to bind mitochondrial Bax (Fig. 3e) and induce dimerization and cytochrome *c* release (Fig. 3f).

As Bax is normally cytosolic, we tested whether 3C10 could also activate WT Bax that was present in cytosolic extracts or that was generated as recombinant protein. Unexpectedly, although 3C10 could bind WT Bax from both sources (Fig. 3g), it failed to trigger Bax translocation to the mitochondria or cytochrome *c* release (Fig. 3h). Notably, 3C10 also prevented tBid-triggered Bax translocation (Fig. 3i) and cytochrome *c* release (Fig. 3j). A similar blockade effect was very recently reported for a Fab that bound residues of α1, α6 and the α1–α2 loop^35^.

### Structural model shows 7D10 binding causes cavities under α1

**Figure 4:**
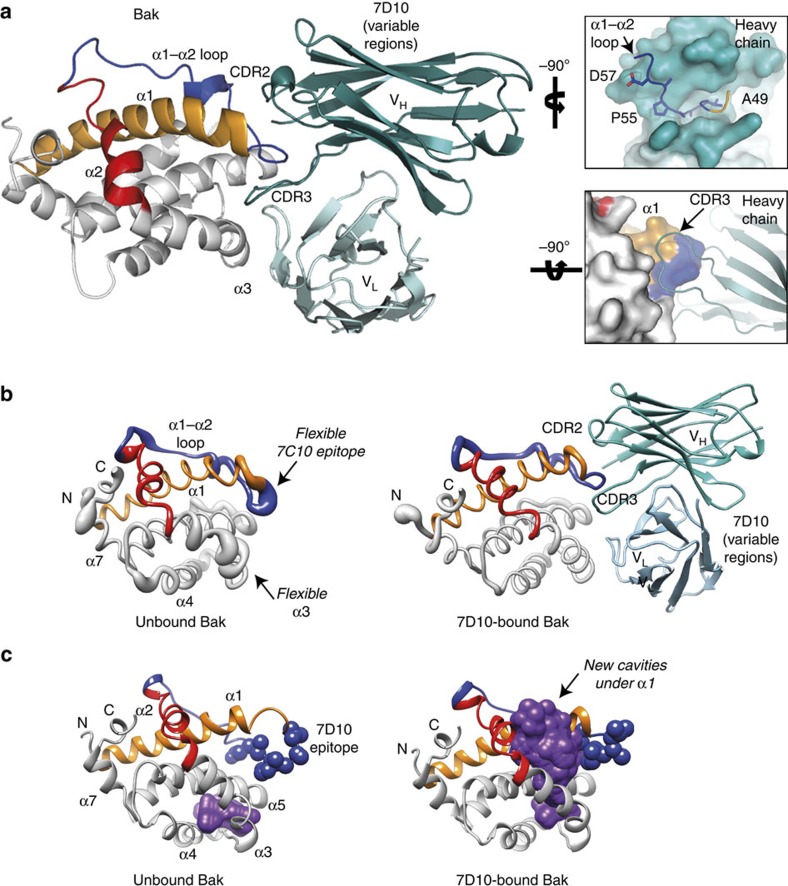
A structural model indicates that 7D10 binds under the α1/loop region to generate cavities underneath α1. (**a**) Final model of the Bak-7D10 complex following docking and MD simulations. Bak is shown as cartoon (white), with α1 (orange), the α1–α2 loop (blue) and α2 (red) highlighted. The 7D10 variable regions are shown as cartoon, with heavy chain (teal) and light chain (cyan) as indicated. Note that the 7D10 epitope is clasped by the CDR2 and CDR3 of the heavy chain (upper insert), with CDR3 inserting in a groove underneath the α1/loop region (lower insert; V_L_ omitted for clarity). (**b**) Flexibility of Bak before and after 7D10 binding from molecular dynamics simulations, displayed in putty format. An ensemble of all structures generated throughout the MD simulation show that before antibody binding (left), the 7D10 epitope and α3 are flexible. Antibody binding (right) selects for a conformation of the epitope belonging to this ensemble of structures with similar energy. Colours as in **a**. (**c**) Molecular dynamics indicate new cavities form between α1 and α2 after 7D10 binds to Bak. The ensemble of all cavities identified throughout the MD simulation is represented as a purple surface, with other colours as in **a**.

The mechanism of antibody-mediated Bak activation was further investigated by generating a molecular model of the 7D10 Fab bound to Bak (Fig. 4). We note that Bak is normally anchored in the outer membrane by α9 forming a transmembrane domain^33^, a feature that may affect Bak activation. However, membranes were not incorporated in the MD simulations as the structure of membrane-bound Bak is not available. Models of Bak obtained from MD simulations were docked with homology-based models of 7D10: several models of both Bak and 7D10, representing different possible loop conformations of each, were cross-docked against one another in an attempt to account for flexibility in the uncomplexed proteins. The final model of the complex after molecular dynamics simulation (Fig. 4a) was also assessed for the effect of antibody binding on the structure of Bak (Fig. 4b,c).

The final model (Fig. 4a) has a large interaction surface (1,411 Å^2^) and high shape (0.670) complementarity. Moreover, the interaction surface reflects our biochemical identification of the 7D10 epitope (Fig. 1d–i) and the availability of cysteine residues at either end of the epitope for linkage in 7D10-activated Bak oligomers (A49C and D57C; Supplementary Fig. 5b–d). Notably, the 7D10 epitope is flanked by CDR2 and CDR3 of the heavy chain. A much smaller contact forms between the light chain and the C terminus of *α*3; this interaction is dominated by weak van der Waals interactions, which may help explain why 7D10 can also bind and activate BaxS184L-GVAAPAD (Fig. 3b,c). Insertion of CDR3 in the groove underneath the α1/loop region may contribute to activation, as both 7D10 and the M2 anti-FLAG antibody failed to activate Bak if the related epitope was positioned ∼11 residues away from α1 (Fig. 2a–c).

Molecular dynamics simulations of non-activated Bak indicated significant flexibility specifically in the region containing the 7D10 epitope and α3 (Fig. 4b, left), consistent with the high B-factors observed in this region in X-ray structures of Bak (2IMT, 2JCN, 2YV6)^36^,^37^. Binding of 7D10 to the epitope resulted in reduced flexibility in both regions (Fig. 4b, right), consistent with 7D10 binding to a preferred conformation. Insertion of the CDR3 loop appears to pry α1 from α2, evidenced by the creation of cavities between these two helices (Fig. 4c, right). In contrast, in unbound Bak only a small cavity is observed between α5 and the α3–α4 loop (Fig. 4c, left), again consistent with the high B-factors in X-ray structures. This weakening of the association of α1 likely represents the initial destabilizing event leading to α1 dissociation from the remainder of Bak (see diagram in Supplementary Fig. 10). Following this event, exposure of the N terminus allows high-affinity interaction of the α1–α2 loop with the heavy chain to persist without hindering formation of BH3:groove dimers (see diagram in Supplementary Fig. 10).

## Discussion

We report here that certain antibodies can bind and activate Bak and mitochondrial Bax to induce cytochrome *c* release. Anti-Bak antibodies required the epitope to be at the start of the α1–α2 loop, and acted by directly dissociating the α1-helix. As antibodies have not been reported to activate either Bak or Bax, nor has the α1–α2 loop region been identified as a trigger site, our findings provide novel opportunities to develop therapeutics that directly activate Bak, and perhaps Bax.

The activation site we have identified at the start of the α1–α2 loop is remote from two sites targeted by BH3-only proteins. The α2–α5 hydrophobic groove in Bak and mitochondrial Bax is engaged by BH3-only proteins to release α1 and unlatch α6–α8 (refs 8, 9, 10, 11, 19). A second site at the α1–α6 rear pocket in Bax (but not Bak) is initially engaged by BH3-only proteins to release α9 from the groove^12^,^13^. The section of the α1–α2 loop bound by three activating antibodies, 7D10, anti-FLAG and 3C10, lies between these two sites.

An intriguing finding was the ability of the 3C10 antibody to trigger cytochrome *c* release via mitochondrial but not cytosolic Bax, whereas tBid released cytochrome *c* via both forms. A partial explanation derived from the ability of 3C10 to block Bax translocation triggered by tBid. Indeed, a recent study reported that Bax translocation triggered by tBid can also be blocked by a synthetic Fab that binds residues in α1, α6 and the α1–α2 loop^35^. The blocking effect of that Fab was attributed to the Fab obstructing tBid from binding to the rear pocket, or suppressing conformational changes in the N-terminal surface^35^. As the rear pocket does not seem to be involved in antibody-triggered activation of mitochondrial Bax (or Bak), 3C10 may be unable to activate cytosolic Bax because it suppresses release of α9 from the Bax groove, or because it more directly prevents Bax from associating with the mitochondrial outer membrane.

While BH3-only proteins do not bind the α1–α2 loop, several non-Bcl-2 proteins reported to directly activate Bak or Bax, may do so. These include Bif-1, αβ amyloid oligomers, protein disulfide isomerases and p53 (refs 15, 18, 38, 39, 40), although p53 has recently been reported to bind to the Bax α6–α9 region^41^. As these proteins lack well-defined BH3 domains, they may bind to the α1–α2 loop, or to a region nearby, to dislodge the α1 helix. It is also possible that proteins may bind to this region to inhibit Bak and Bax activation, as proposed for the Bcl-2 BH4 fragment^42^, and act in a similar way to tethering the α1–α2 loop within Bak (Fig. 2d,e) or within Bax^12^.

Bak conformational changes induced by antibody (see diagram in Supplementary Fig. 10) are similar to those induced by BH3-only proteins (see diagram in Supplementary Fig. 6c), as both agents induced similar Bak conformational change and oligomerization, and cytochrome *c* release initiated by either agent was blocked by each of four tethers within Bak. However, the initial unfolding events triggered by both agents were different, consistent with distinct binding sites on Bak. tBid binding first initiates movement of α2 (Supplementary Fig. 6c)^8^,^19^, consistent with binding to the α2–α5 groove. In contrast, 7D10 binding initially triggers α1 dissociation, as 7D10 and FLAG epitopes allowed activation only when placed proximal to α1, and tethering the loop to α1 did not block 7D10-triggered Bak activation. Moreover, molecular dynamics of a 7D10:Bak structural model caused extrication of α1 from α2 to form cavities between the two helices.

As either Bak or Bax is essential for mitochondrial apoptosis, developing agents that directly trigger their activation is of great interest^43^,^44^. Developing anti-cancer agents that target the Bak loop may complement BH3-mimetics that target the Bcl-2 prosurvival proteins^1^,^45^. While BH3-mimetics release endogenous BH3-only proteins that activate both Bak and Bax^46^,^47^,^48^, the 7D10 antibody, and other activators that might directly dislodge α1, are likely to specifically bind Bak due to the lack of sequence homology in the loop regions. This specificity also implies that this class of activators cannot be sequestered by prosurvival Bcl-2 proteins such as Bcl-x_L_ and Mcl-1, and could bypass resistance caused by those proteins.

It remains to be demonstrated that the 7D10 Fab-induced changes in oocyte mitochondrial morphology are due to Bak conformational change. There are very few studies investigating apoptosis in oocytes at the immature fully grown germinal vesicle stage we have used. It is well known that early oocytes in primordial follicles undergo a TAP63-mediated apoptosis in response to DNA damage^49^ but downregulation of TAP63 on the initiation of oocyte growth is thought to make fully grown oocytes, such as those used here, more refractory to apoptosis^50^. Some evidence of oocyte fragmentation to form apoptotic bodies has been reported when mature ovulated oocytes are allowed to age *in vitro* but the outward signs of apoptosis remain controversial at these mature stages^51^,^52^. The limited availability of healthy human oocytes for these studies is a practical limitation on our ability to formally prove the association of mitochondrial morphology with Bak conformational change.

In conclusion, we have identified the proximal α1–α2 loop as a second activation site in Bak and in mitochondrial Bax. This site can be targeted by antibodies to directly dissociate α1, and as a result induce the same Bak oligomerization and pore formation that is induced by BH3-only proteins. This site may allow the development of novel therapeutics such as intrabodies based on 7D10, and possibly small molecules, that pry α1 from α2. Unlike the BH3-mimetics, such agents would specifically activate either Bak or Bax, and, as they would not be sequestered by prosurvival Bcl-2 proteins, they may bypass resistance due to those proteins. Conversely, agents that bind the α1/loop region and prevent α1 dissociation may provide novel anti-apoptotic agents.

## Methods

### Cloning and expression of Bak and Bax variants

Bak and Bax mutants were generated by site-directed mutagenesis and overlap extension PCR using Phusion High-Fidelity Master Mix (New England BioLabs). The two BaxS184L variants also contain two cysteine substitutions in the BH3 domain (S55C) and groove (R94C) to monitor BH3:groove interaction after Bax activation and oligomerization^53^. Primer sequences are listed in Supplementary Table 1. PCR products were digested with EcoRI and XhoI, and cloned into the pMX-IRES-GFP retroviral expression vector, and the introduced mutations confirmed by DNA sequencing. The variants were then stably expressed in SV40-immortalized *Bak*^*−/−*^*Bax*^*−/−*^ mouse embryonic fibroblasts (MEFs), and were a gift from Professor David Huang. MEFs were grown in Dulbecco's modified Eagle's medium (DMEM) supplemented with 10% fetal bovine serum, 0.1 mM L-asparagine and 55 μM 2-mercaptoethanol. All cultures were maintained at 37 °C in a humidified 10% CO_2_ incubator.

### Cell death assays

For each cell line expressing a Bak or Bax variant, 20,000 and 80,000 cells were seeded in a six-well plate and on the following day wells seeded with 80,000 cells were incubated with 10 μM etoposide for 24 h to induce cell death. Cells were collected and resuspended in KDS-BSS buffer and 5 μg μl^−1^ propidium iodide (PI), and cell death (%PI-positive) assessed by flow cytometry.

### Preparation of mitochondrial fractions

Mitochondria-enriched membrane fractions were generated by resuspending MEFs at 1 × 10^7^ ml^−1^ in MELB buffer (93.5 mM sucrose, 20 mM HEPES, pH 7.4, 2.5 mM MgCl_2_ and 100 mM KCl) supplemented with Complete Protease Inhibitor cocktail (Roche) and 0.025% w/v digitonin to permeabilize the cell membrane. Cells were left on ice for 10 min and centrifuged at 13,000 *g* for 5 min to separate the supernatant (cytosolic) and pellet (mitochondria-enriched membrane) fractions, and the membrane fractions resuspended in MELB buffer with protease inhibitors.

### Mitochondrial incubations and cytochrome *c* release assays

For activation of Bak or BaxS184L and mitochondrial cytochrome *c* release, 50 μl of membrane fractions were incubated with 100 nM caspase-8-cleaved human Bid (tBid)^54^, or with the indicated antibody (5 μg) for 30 min at 30 °C. Activation of WT Bax expressed in cells or as recombinant protein^9^ (Fig. 3h,i) is described in the figure legends. To monitor cytochrome *c* release from mitochondria, reactions were spun at 13,000 *g* and the supernatant and pellet fractions immunoblotted for cytochrome *c*.

### Bak activation assessed by limited proteolysis

Bak conformational change was assessed by limited proteolysis^55^. Briefly, 50 μl of membrane fractions resuspended in MELB with 4 μg ml^−1^ pepstatin A (Sigma) were pre-chilled to 0 °C then incubated with 30 μg ml^−1^ proteinase K (Roche) or trypsin (Sigma) on ice for 20 min, or 0.06 U μl^−1^ enterokinase (Merck Millipore) at room temperature for 2 h. The reactions were stopped by addition of 1 mM phenylmethylsulfonyl fluoride (PMSF) and immunoblotted with antibodies to the Bak BH3 domain (4B5).

### Disulfide bond formation in Bak

Bak conformational change and oligomerization was assessed by inducing disulfide bonds within or between Bak molecules. Membrane fractions were exposed to the redox catalyst copper (II)(1,10-phenanthroline)_3_ (CuPhe) diluted 100-fold from a stock containing 30 mM CuSO_4_ and 100 mM 1,10-phenoanthroline in 4:1 water:ethanol^56^. Reactions were incubated for 30 min on ice and then re-suspended in SDS sample buffer containing 20 mM EDTA before analysis by non-reducing SDS–PAGE and immunoblotting. Where indicated, CuPhe was also used to ‘tether' non-activated Bak by forming disulfide bonds between cysteine residues^19^. Briefly, fractions were incubated with CuPhe (1 mM) for 5 min on ice followed by quenching with 20 mM *N*-ethylmaleimide (NEM) for 10 min at room temperature.

### Immunoprecipitation

Briefly, whole cell or membrane fractions from 3–5 × 10^6^ cells were solubilized with 1% w/v digitonin in lysis buffer (20 mM Tris, 135 mM NaCl, 1.5 mM MgCl_2_, 1 mM EGTA, 10% glycerol, pH 7.4) for 1 h on ice. Lysates were centrifuged at 13,000 *g* for 5 min and pre-cleared with 50 μl Protein G sepharose (Amersham Biosciences). Pre-cleared supernatant was then incubated with antibody (4 μg) and Protein G sepharose. Unbound proteins were collected and the resin washed with lysis buffer containing up to 0.1% w/v digitonin. Immunoprecipitated proteins (IP) were eluted by boiling in sample buffer, and together with unbound (UB) and total lysates (input), were analysed by immunoblotting. Several rat monoclonal antibodies generated in-house were used for immunoprecipitation, including clones 8F8 and 7D10 that recognize Bak^22^,^23^, and clone 3C10 that recognizes Bax (Fig. 3d). Other antibodies include mouse monoclonal anti-FLAG (M2; Sigma) and several anti-Bak antibodies described in Supplementary Fig. 2.

### SDS–PAGE and western blotting

Samples were resolved by SDS–PAGE (Bio-Rad), and transferred to 0.22 μm nitrocellulose or polyvinylidene difluoride membranes. Primary antibodies used were rabbit polyclonal anti-Bak aa23-38 (1:5,000, #B5897; Sigma), rat monoclonal anti-Bak that recognizes Bak aa82-86 (1:2,000, clone 4B5, R. Kluck), rat monoclonal anti-Bak that recognizes Bak aa51-55 (1:2,000, clone 7D10, D.C. Huang), rat monoclonal anti-Bax that recognizes Bax aa12-24 (1:5,000, clone 49F9, D.C. Huang), rabbit polyclonal anti-Bax NT (1:1,000, #ABC11; Millipore), mouse monoclonal anti-cytochrome *c* (1:2,000, #556433; BD Pharmingen) and mouse monoclonal anti-VDAC (1:2,000, #AB10527, Merck Millipore). Detection was achieved using horseradish peroxidase (HRP)-conjugated anti-rabbit (1:5,000, #4010-05, Southern Biotech), anti-rat (1:5,000, #3010-05, Southern Biotech) and anti-mouse (1:2,000, #1010-05, Southern Biotech) secondary antibodies. To avoid signals from antibody light chains in western blots, heavy chain-specific HRP-conjugated goat anti-rabbit IgG (1:5,000, #4041-05; Southern Biotech) and goat anti-rat IgG (1:5,000, #3030-05; Southern Biotech) were also used. To identify Fab fragments, HRP-conjugated goat anti-rat light chain-specific IgG (1:2,000, #112-035-175; Jackson ImmunoResearch) was used. Proteins were visualized by Luminata Forte Western HRP substrate (#WBLUF0500, Millipore) on a ChemiDoc XRS+System, and images processed with ImageLab Software (Bio-Rad). Uncropped images from Figs 1, 2, 3 are shown in Supplementary Figs 11–13.

### Bax membrane integration

To assess Bax membrane integration, membrane fractions were subjected to carbonate extraction. Mitochondria isolated from *Bak*^*−/−*^ mouse liver were incubated for 2 h at 37 °C with 50 nM recombinant Bax, and with tBid and 3C10 as indicated. The incubations were centrifuged at 13,000 *g* for 5 min to obtain cytosolic and pellet fractions. The pellet was re-suspended in 0.1 M Na_2_CO_3_ (pH 11.5) and incubated on ice for 20 min. pH was neutralized with an equal volume of 0.1 M HCl and the sample incubated at 22 °C for 5 min. Samples were then supplemented with nuclease buffer from a 10 × stock (400 mM Tris, 100 mM MgSO_4_, 10 mM CaCl_2_) and 1 U DNase, before being centrifuged at 13,000 *g* for 10 min, and supernatant (peripherally attached) and pellet (membrane inserted) fractions immunoblotted for Bax.

### Generation and purification of 7D10 Fab

The 7D10 antibody was cleaved with papain to generate the Fab fragment. Papain (Sigma) at 1 mg ml^−1^ was solubilized and activated in 10 mM cysteine and 20 mM EDTA in PBS for 10 min on ice. Following 10-fold dilution in PBS, papain was added to the 7D10 antibody at a ratio of 1:20 and incubated at 37 °C followed by inactivation with 30 mM iodoacetamide (Sigma). Papain-cleaved antibody was dialysed overnight in Buffer A (10 mM acetic acid, pH 4.5) and applied to a Mono S column equilibrated in Buffer A. A linear 20 ml gradient with Buffer B (10 mM acetic acid, 500 mM NaCl, pH 4.5) was used to separate Fab and Fc in 0.5 ml fractions.

### Microinjection of 7D10 Fab

Human germinal vesicle (GV) staged oocytes were collected from Monash IVF Australia under ethics approval number CF13/2664-2013001409 provided by the Monash University Human Research Ethics Committee. Oocytes were incubated in M2 media (Sigma-Aldrich) containing 20 nM tetramethylrhodamine methyl ester (TMRM) (Sigma-Aldrich) at 37 °C for 30 min. Oocytes were then either microinjected^57^ with Fab from 7D10, or with control Fab from the rat monoclonal 9E10 anti-c-Myc antibody (kindly provided by J. Menting), and imaged live using confocal microscopy (Leica SP8) every 10 min for 3 h.

### Mice oocyte collection and culture

GV oocytes were collected from 4- to 6-week-old female C57BL/6 mice that had been administered 10 international units pregnant mare's serum gonadotropin (PMSG; Intervet) by intraperitoneal injection 48 h earlier. Cumulus-enclosed GV-stage oocytes were recovered by mechanical perforation of the ovaries with a 27-gauge needle. The cumulus cells were removed by repeated pipetting using narrow-bore glass Pasteur pipettes. Oocytes were placed into M2 medium (Sigma-Aldrich) at a temperature of 37 °C. GV arrest was maintained where necessary by the addition of 200 μM 3-isobutyl-1-methylxanthine (IBMX; Sigma-Aldrich) to the medium. Mouse oocytes were microinjected similar to the human oocytes. All animal experiments were performed under ethics approval number MARP/2012/143 provided by the Monash University Animal Research Ethics Committee, and according to approved guidelines.

### Image analysis

Accurately scaled single slice oocyte images at 3 h post injection were converted using ImageJ to 8-bit then a threshold was applied accordingly to each image. An ROI around each oocyte was then used to analyse the number and size of particles.

### Molecular dynamics and protein–protein docking

Computational docking of Bak and 7D10 was performed using structures of 7D10 obtained from the RosettaAntibody3 online server^58^, and structures of Bak obtained from molecular dynamics (MD) simulations. The sequence of 7D10 is available from International Patent Application No. PCT/AU2015/000290. Flexibility (beyond just side-chain movement) was incorporated in the docking by considering multiple structures of both Bak and 7D10.

Molecular dynamics simulations used the GROMACS (v5.0.4) package of programs^59^ with the OPLS-aa force field^60^. Ionizable residues were assumed to be in their charged state. Each molecule was solvated in a water box extending 10 Å beyond all atoms; sodium and chloride ions were added to neutralize the system and provide a final ionic strength of 0.1 M. Protein and water (with ions) were coupled separately to a thermal bath at 300 K using velocity rescaling^61^ applied with a coupling time of 0.1 ps, whereas the pressure was coupled to an isotropic barostat using a time constant of 1 ps and compressibility of 4.5 × 10^5^ per bar. All simulations were performed with a single non-bonded cutoff of 10° and applying a neighbour-list update frequency of 10 steps (20 fs). The particle-mesh Ewald method was used to account for long-range electrostatics^62^ (applying a grid width of 1.2 Å and a fourth-order spline interpolation). Bond lengths were constrained using the LINCS algorithm^63^. All simulations consisted of an initial minimization of water molecules, followed by 100 ps of MD with the protein restrained. Following positional restraints, all restraints on the protein were removed and MD continued for a further 200 ns. Coordinates were archived throughout the simulation at 10 ns intervals—these structures were used in subsequent docking calculations. The models of the 7D10 antibody Fv region included the original homology model and 10 top-scoring models from H3 loop refinement.

The ZDOCK program^64^ was used to dock 21 structures of Bak to 11 structures of the 7D10 Fv with 6° rotational sampling of the ligand. Docking to 7D10 was limited to the CDR loops (including three residues on either side of each loop). The Bak α8-helix was blocked from participating in the interface since its proximity to the membrane should prevent antibody binding, thus providing a weak constraint on the likely orientation of Bak relative to the membrane. Subsequent re-scoring of the top-scoring 2,000 results from each of the 231 ZDOCK calculations was conducted using the ZRANK (v2) program^65^. The top-scoring 2,000 complexes following ZRANK rescoring, inclusive of all 462,000 complexes, were refined using the RDOCK program^66^. The top-scoring 20 complexes from refinement using RDOCK were manually examined for consistency with experimental data. Shape complementarity of the top-scoring 20 RDOCK refined structures was conducted using the Sc program^67^.

MD simulations were performed on both the unbound Bak and the final Bak-7D10 complex obtained from the RDOCK analysis; these simulations were each run for 100 ns. Cavity analysis was performed using the trj_cavity tool^68^.

### Data availability

The authors declare that all data supporting the findings of this study are available within the article and its Supplementary Information files.

## Additional information

**How to cite this article:** Iyer, S. *et al*. Identification of an activation site in Bak and mitochondrial Bax triggered by antibodies. *Nat. Commun.* 7:11734 doi: 10.1038/ncomms11734 (2016).

## Supplementary Material

Supplementary Information Supplementary Figures 1-13 and Supplementary Table 1.
