# Supplementary material for: Identification of an activation site in Bak and mitochondrial Bax triggered by antibodies
Source: Nat Commun. 2016 May 24;7:11734. doi: 10.1038/ncomms11734 (PMC4890306; doi:10.1038/ncomms11734)
Supplement: Supplementary Information — Supplementary Figures 1-13 and Supplementary Table 1. [file ncomms11734-s1.pdf]

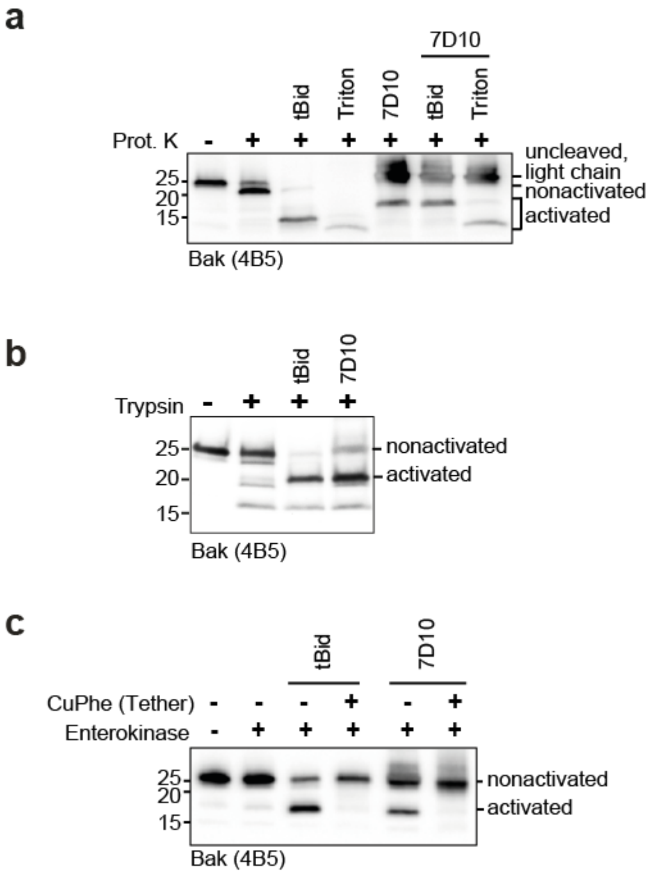

Supplementary Figure 1. 7D10 triggers Bak conformation change as indicated by increased susceptibility to proteinase K, trypsin and enterokinase.

(**a**) Proteinase K cleaves Bak after its activation by either tBid or 7D10, but 7D10 masks one of the cleavage sites. Membrane fractions from *Bak*$^{-/-}$*Bax*$^{-/-}$ MEFs expressing hBak were incubated with tBid, 1% v/v Triton-X100 or the 7D10 antibody, before incubation with proteinase K and immunoblotting for Bak. Note that in lane 6, 7D10 addition after incubation with tBid masks a cleavage site.

(**b**) Trypsin cleaves Bak after its activation by either tBid or 7D10. Membrane fractions incubated with tBid or 7D10 as in (a) were incubated with trypsin and immunoblotted for Bak.

(**c**) Enterokinase cleaves Bak after its activation by either tBid or 7D10. Membrane fractions incubated with tBid or 7D10 as in (a) were incubated with enterokinase and immunoblotted for Bak. In two samples pre-incubated with the oxidant CuPhe to induce a C14:C166 tether in hBak (see Supplementary Fig. 6a), Bak did not become susceptible to enterokinase cleavage.

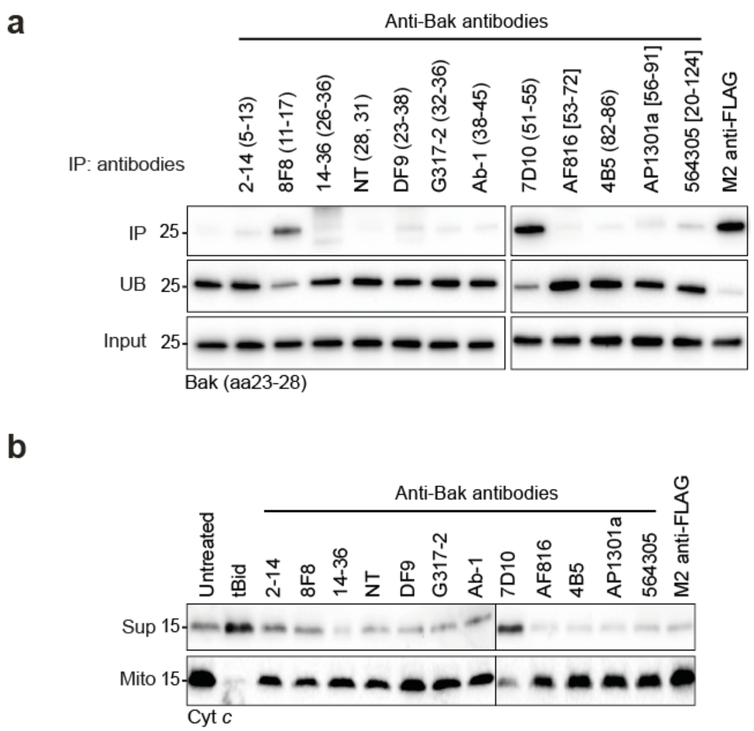

Supplementary Figure 2. The 7D10 antibody, but not other Bak antibodies, can trigger Bak conformation change.

(**a**) Binding of three antibodies to non-activated Bak. Membrane fractions from *Bak*$^{-/-}$*Bax*$^{-/-}$ MEFs expressing FLAG-tagged human Bak were immunoprecipitated with the indicated antibodies, followed by immunoblotting for Bak. The epitopes in the hBak sequence (Alsop et al., 2015) are indicated in round brackets. Alternatively, the sequences used as immunogens for three antibodies, Cat#AF816 (Novus Biologicals), Cat#AP1301a (Abgent) and Clone 564305 (R&D Systems) are indicated in square brackets.

(**b**) Only the 7D10 antibody activates Bak to release cytochrome *c*. Membrane fractions from (a) were incubated with tBid or with the indicated antibodies for 30 min at 30°C and assessed for cytochrome *c* release.

Data in (a) and (b) are representative of two independent experiments.

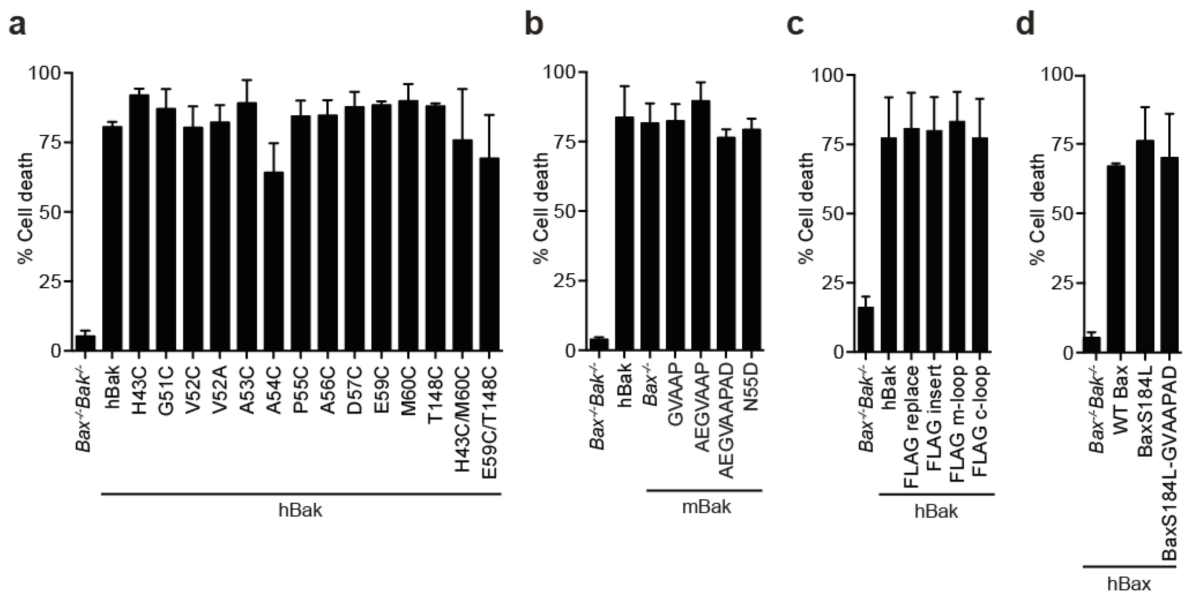

Supplementary Figure 3. Bak and Bax α1-α2 loop variants retain pro-apoptotic function. (**a-d**) *Bak*$^{-/-}$*Bax*$^{-/-}$ MEFs stably expressing the indicated Bak or Bax variants were untreated or treated with etoposide (10 μM) for 24 h and cell death assessed by propidium iodide uptake. In (**b**) WT mouse Bak was tested using *Bax*$^{-/-}$ MEFs.

Data are mean and s.d. of three independent experiments.

Note that as each variant was able to mediate etoposide-triggered cell death, as well as tBid-mediated cytochrome *c* release (Figs. 1e and i, 2c, 3c), at least a portion of each variant was able to convert from the non-activated to the activated conformation and generate pores. However, we cannot exclude the possibility that variants may have slightly more or less function than wild type Bak or Bax**.**

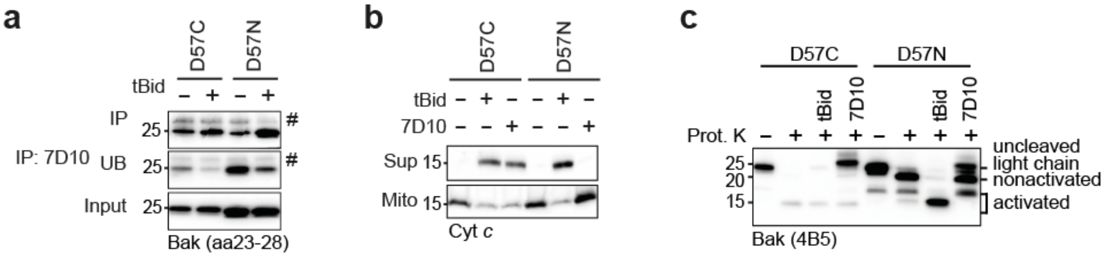

Supplementary Figure 4. The D57N substitution in human Bak inhibits binding and activation by 7D10 antibody.

(**a**) The D57N substitution in human hBak inhibits binding of 7D10. Membrane fractions from *Bak*$^{-/-}$*Bax*$^{-/-}$ MEFs expressing the indicated hBak variants were incubated with or without tBid followed by immunoprecipitation with 7D10 and immunoblotting for Bak. IP, immunoprecipitated; UB, unbound; #, light chain.

(**b**) The D57N substitution in human hBak inhibits 7D10-induced cytochrome *c* release. Membrane fractions from (a) were incubated with tBid or 7D10 and assessed for cytochrome *c* release.

(**c**) The D57N substitution in human hBak inhibits 7D10-induced conformation change. Incubations from (b) were incubated with proteinase K as in Fig. 1b, and show minimal cleavage. Note that even prior to activation, the D57C variant was susceptible to cleavage (see Supplementary Fig. 5a).

Data are representative of three independent experiments.

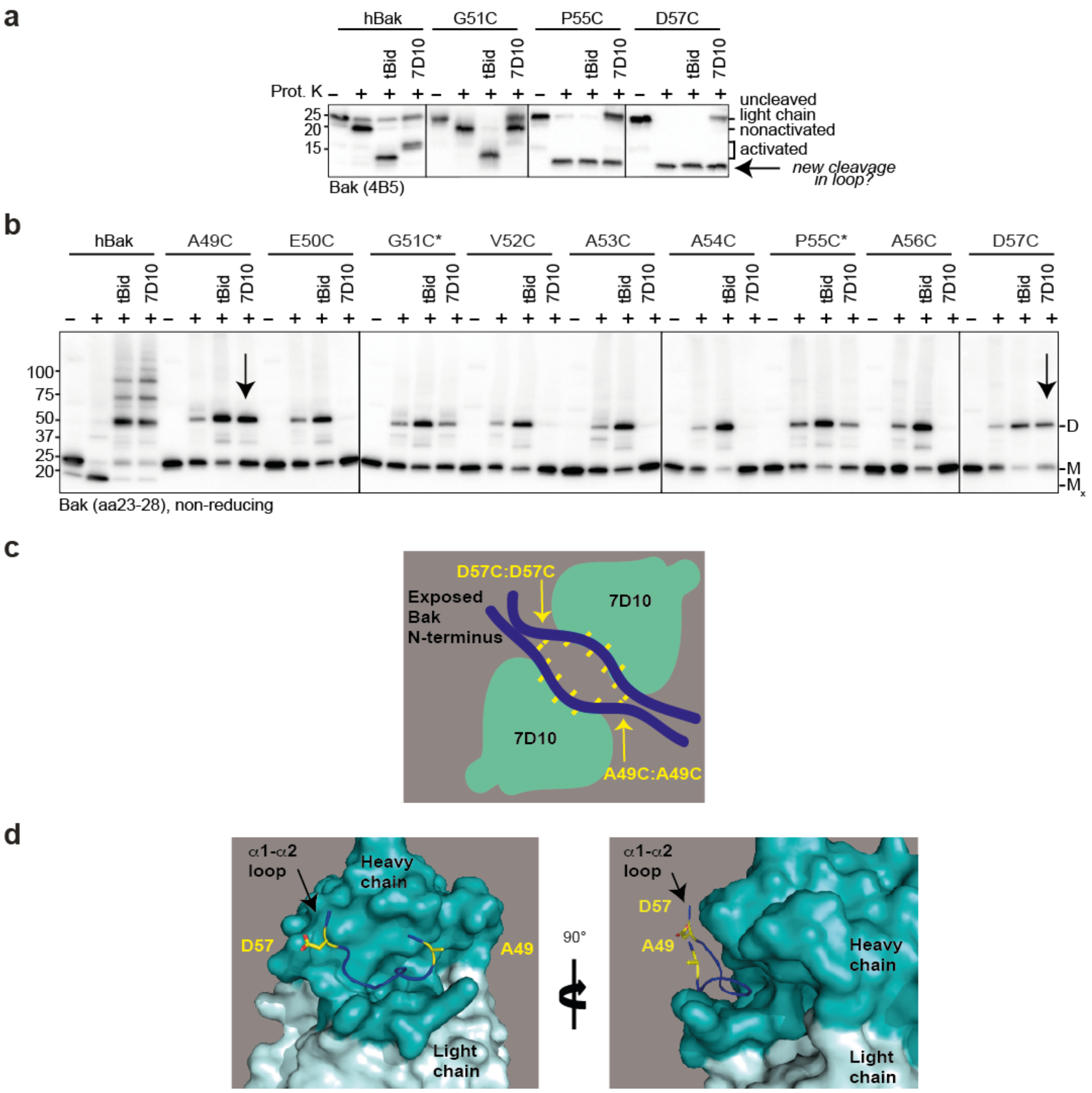
a
hBak
G51C
P55C
D57C
tBid
7D10
Prot. K
25
20
15
uncleaved
light chain
nonactivated
activated
new cleavage in loop?
Bak (4B5)
b
hBak
A49C
E50C
G51C*
V52C
A53C
A54C
P55C*
A56C
D57C
100
75
50
37
25
20
D
M
Mx
Bak (aa23-28), non-reducing
c
D57C:D57C
Exposed Bak N-terminus
7D10
7D10
A49C:A49C
d
α1-α2 loop
Heavy chain
D57
A49
Light chain
90°
α1-α2 loop
D57
A49
Heavy chain
Light chain

Supplementary Figure 5. Characterization of the 7D10 epitope.

(**a**) The P55C and D57C mutations alter Bak cleavage by proteinase K. Membrane fractions from *Bak*$^{-/-}$*Bax*$^{-/-}$ MEFs expressing the indicated hBak variants were treated with tBid or 7D10 prior to treatment with proteinase K. Note that even prior to activation, the P55C and D57C variants were susceptible to cleavage, possibly due to a change in the structure of the α1-α2 loop.

(**b**) The 7D10-binding site on Bak lies between residues A49 and D57. Membrane fractions treated as in Fig. 1c were incubated with oxidant (CuPhe) and immunoblotted for Bak. Note that after activation by tBid, cysteine residues at positions A49 to D57 (arrows) can each disulfide bond between Bak molecules (dimers, D), as depicted in (c). In contrast, after activation of each variant by 7D10 (* except for G51C and P55C which do not get activated; Fig. 1e,f), cysteine residues at positions E50, V52, A53, A54 and A56 cannot disulfide bond, presumably due to the 7D10 antibody. Hence, as A49C and D57C can still link to dimers, they are external to the 7D10 epitope.

(**c**) Schematic of the 7D10-binding site on Bak, illustrating substituted cysteine residues in (b) that can and cannot link between activated Bak molecules when 7D10 is bound.

(**d**) Model of the Bak-7D10 complex (also see Fig. 4), highlighting the availability of A49C and D57C for disulfide bonding between activated Bak molecules, even when 7D10 is bound to activated Bak.

Data in (a) and (b) are representative of three independent experiments.

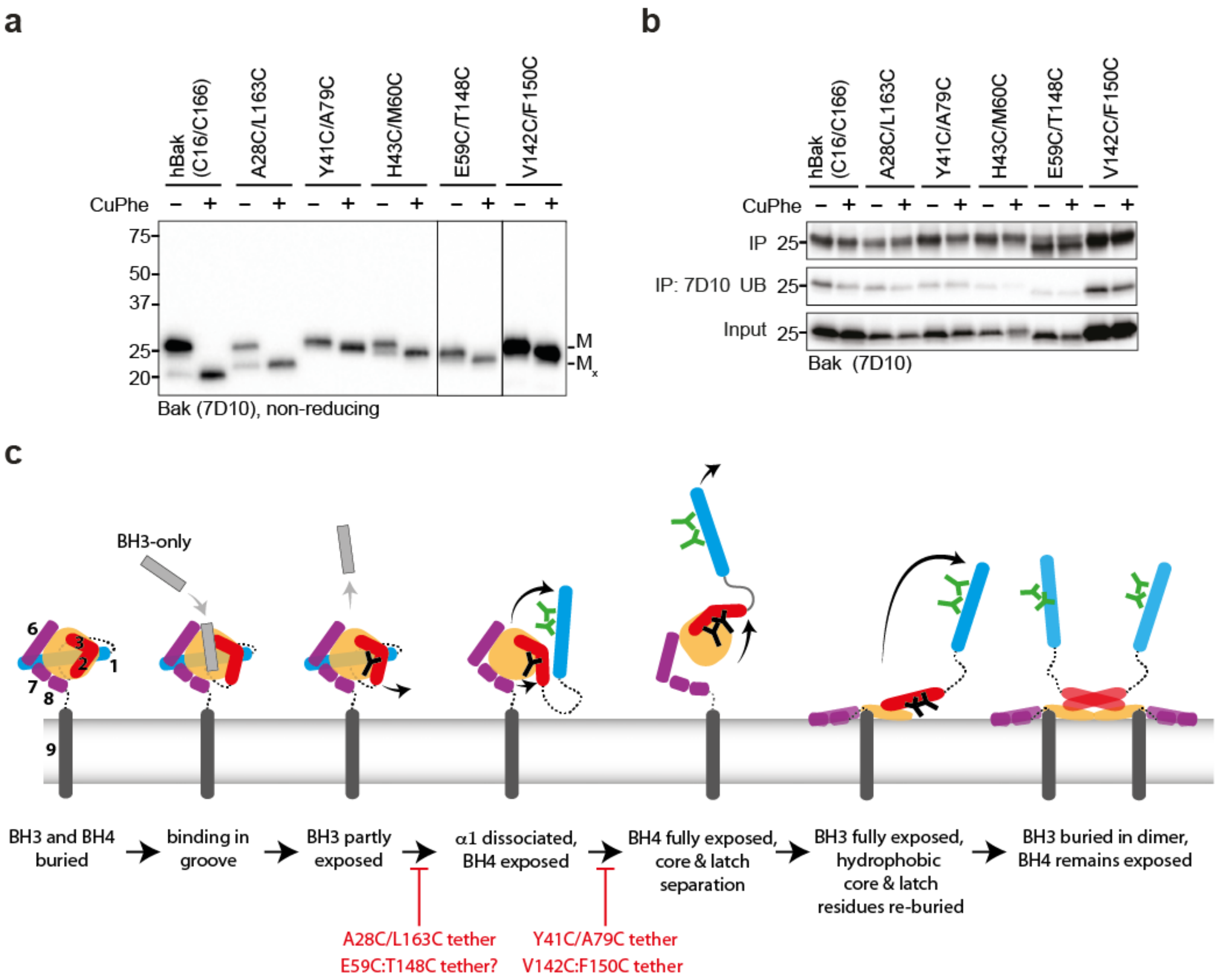

Supplementary Figure 6. Intramolecular tethers in Bak are efficient and do not prevent binding of 7D10 to Bak.

(**a**) Disulfide bonds within Bak are near complete after addition of oxidant. Membrane fractions from *Bak*$^{-/-}$*Bax*$^{-/-}$ MEFs expressing the indicated double cysteine variants were incubated with oxidant (CuPhe), resolved on non-reducing SDS-PAGE, and immunoblotted for Bak. M, monomer; M_x, intramolecular tether.

(**b**) 7D10 binds to all tethered forms of Bak. Membrane fractions from (a) were immunoprecipitated with 7D10 and immunoblotted for Bak.

(**c**) Effect of tethers on Bak conformation change. Approximate steps at which the tethers block conformation changes induced by tBid, based on epitope exposure [1], although the E59C:T148C tether was not tested in those studies. The α1 (blue), core (orange/red), latch domain (purple) and transmembrane domain (dark grey) are illustrated. Y indicates an epitope is partially accessible to antibody, while YY indicates an epitope is fully accessible to antibody. Adapted from Alsop et al, Nat Commun 6, 6841 (2015).

Data in (a) and (b) are representative of two independent experiments.

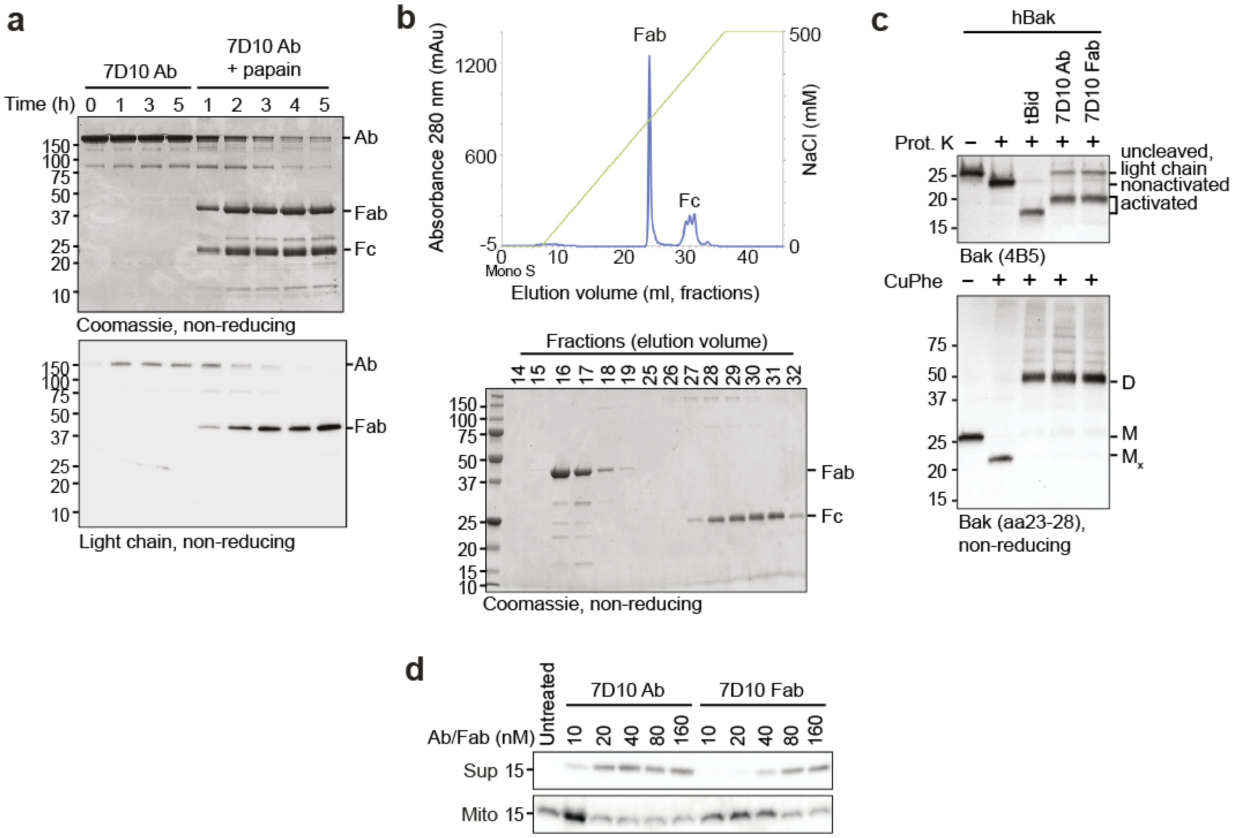

Supplementary Figure 7. The 7D10 Fab also activates Bak.

(**a**) Cleavage of 7D10 generates the Fab fragment. The 7D10 antibody was incubated with papain and run on non-reducing SDS-PAGE followed by Coomassie staining (top panel) or by immunoblotting for light chain (lower panel).

(**b**) Purification of Fab. Papain-digested 7D10 was run on Mono S ion exchange chromatography (upper panel) and the indicated fractions analyzed by Coomassie staining (lower panel). The NaCl gradient is indicated by the sloping line (green).

(**c**) The 7D10 Fab induces Bak conformation change and oligomerization. Membrane fractions from $Bak^{-/-}Bax^{-/-}$ MEFs expressing Bak were incubated with tBid, 7D10 or 7D10 Fab. Aliquots were then incubated with proteinase K (upper panel) or oxidant (CuPhe, lower panel) followed by immunoblotting for Bak. D, linked dimers; M, monomers, $M_{X;}$ intramolecular-linked monomers.

(**d**) 7D10 antibody and Fab show similar cytochrome *c* release. Membrane fractions were incubated as in (c) with the indicated concentrations of 7D10 antibody and Fab, and analysed for cytochrome *c* release.

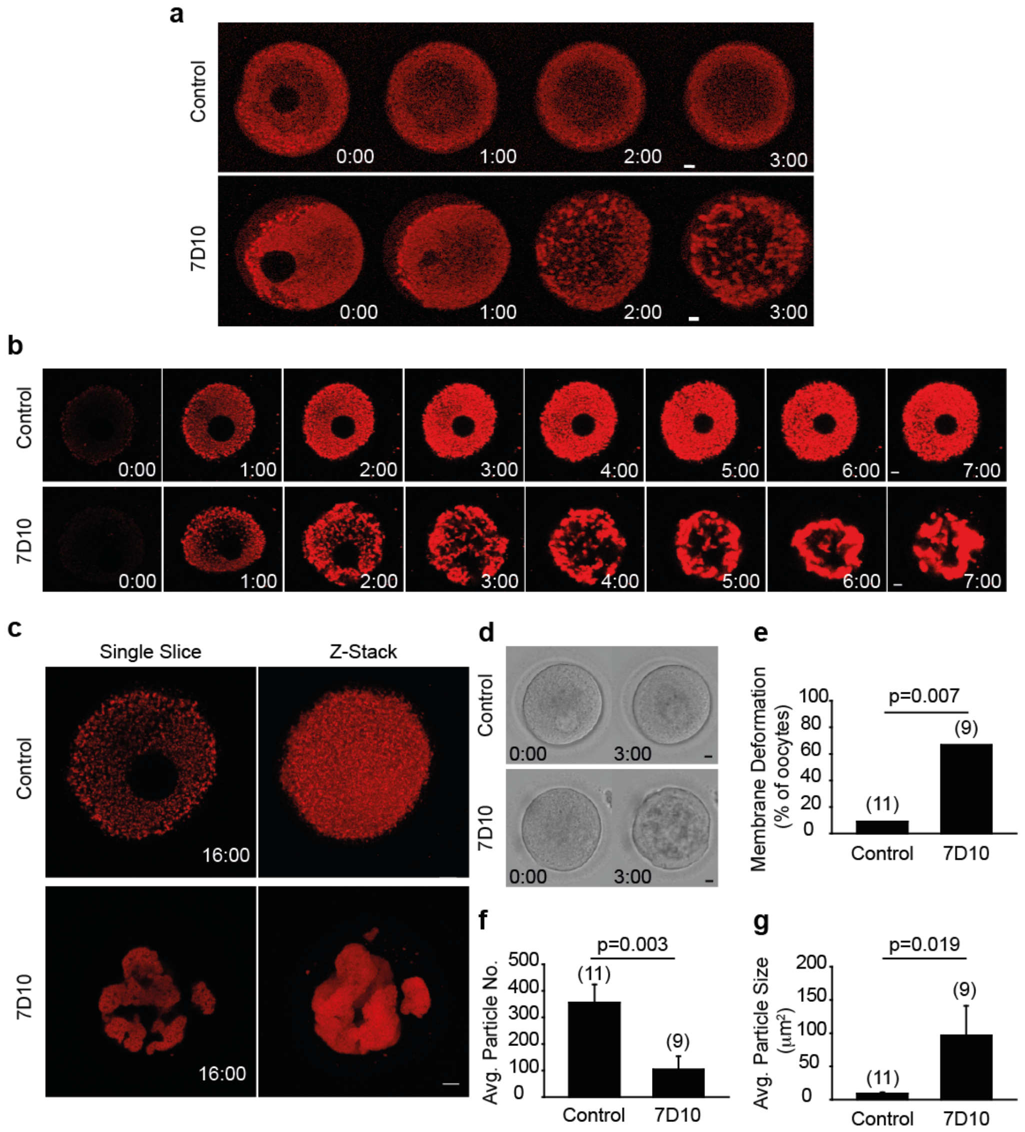

Supplementary Figure 8. 7D10 Fab causes abnormal mitochondrial aggregation in human oocytes.

(**a**) Representative live cell images of human oocytes injected with control or 7D10 Fab fragment for 3 h, showing mitochondrial aggregation.

(**b**) Live cell imaging of human oocytes injected with control or 7D10 Fab fragment for 7 h, showing increased aggregation past 3 h.

(**c**) High resolution images taken with a 63x objective with optical zoom of the oocytes shown in (b) at 16 h post injection, revealing detailed mitochondria morphology in both single slice and Z-stack planes.

(**d**) Brightfield images of control and 7D10 oocytes showing plasma membrane deformation at 3 h post injection.

(**e**) Percentage of oocytes with membrane deformation in control and 7D10 oocytes at 3 h post injection. p=0.007, chi-square test.

(**f**) Average number of particles in individual control and 7D10 oocytes at 3 h post injection. p=0.003, student's t-test.

(**g**) Average particle size in individual control and 7D10 oocytes at 3 h post injection. p=0.028, student's t-test. Scale bars represent 10 µm.

Error bars represent s.e. Time stamps indicate hh:mm. Scale bars represent 10 µm.

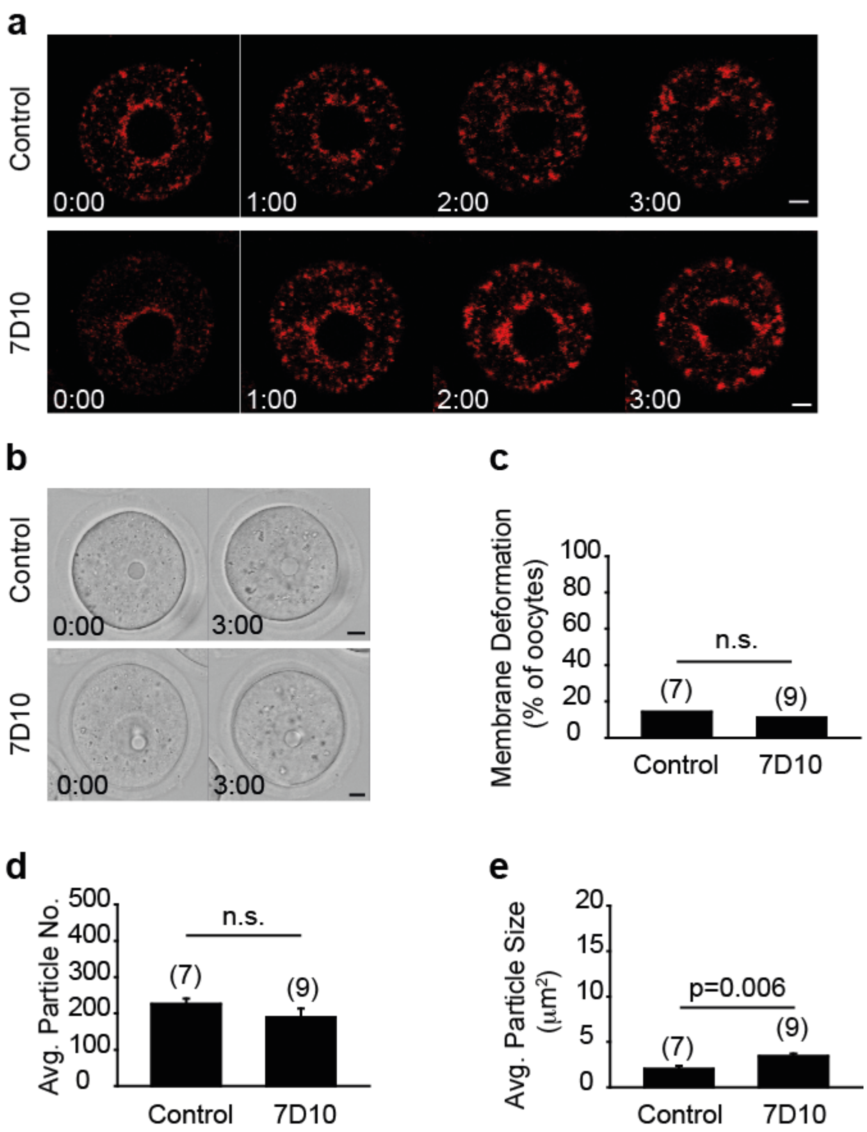

Supplementary Figure 9. 7D10 Fab does not cause abnormal mitochondrial aggregation in mouse oocytes.

(**a**) Live cell imaging of mouse oocytes injected with control or 7D10 Fab fragment for 3 h, showing similar morphology.

(**b**) Brightfield images of control and 7D10 mouse oocytes showing no plasma membrane deformation at 3 h post injection.

(**c**) Percentage of oocytes with membrane deformation in control and 7D10 oocytes at 3 h post injection. $p>0.05$, chi-square test.

(**d**) Average number of particles in individual control and 7D10 oocytes at 3 h post injection. $p>0.05$, student's t-test.

(**e**) Average particle size in individual control and 7D10 oocytes at 3 h post injection. $p=0.006$, student's t-test.

Error bars represent s.e. Time stamps indicate hh:mm. Scale bars represent 10 μm.

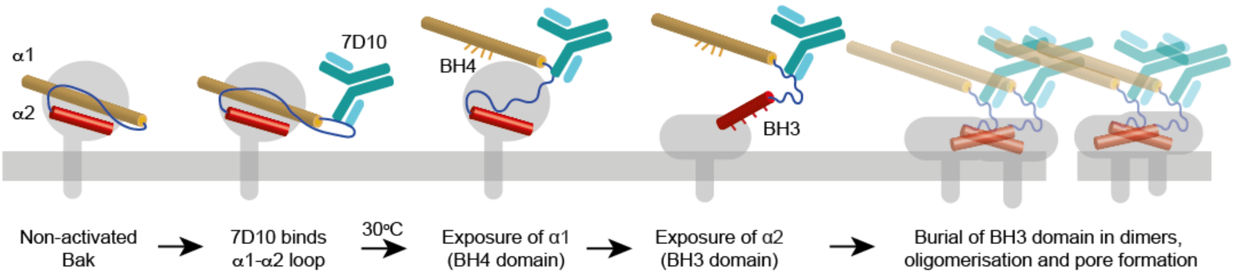

Supplementary Figure 10. Schematic of Bak activation by the 7D10 antibody.
Binding of the 7D10 antibody (teal/cyan) to the Bak α1-α2 loop (blue) close to α1 (orange) directly destabilizes α1 (exposing the BH4 domain). The α1 dissociation allows global conformation changes including exposure of α2 (red; BH3 domain), core/latch separation and their collapse onto the mitochondrial outer membrane surface. Subsequent burial of the BH3 domain in BH3:groove dimers is followed by high order oligomerization and pore formation. For a more detailed diagram of conformation changes induced by tBid, see Supplementary Fig. 6c.

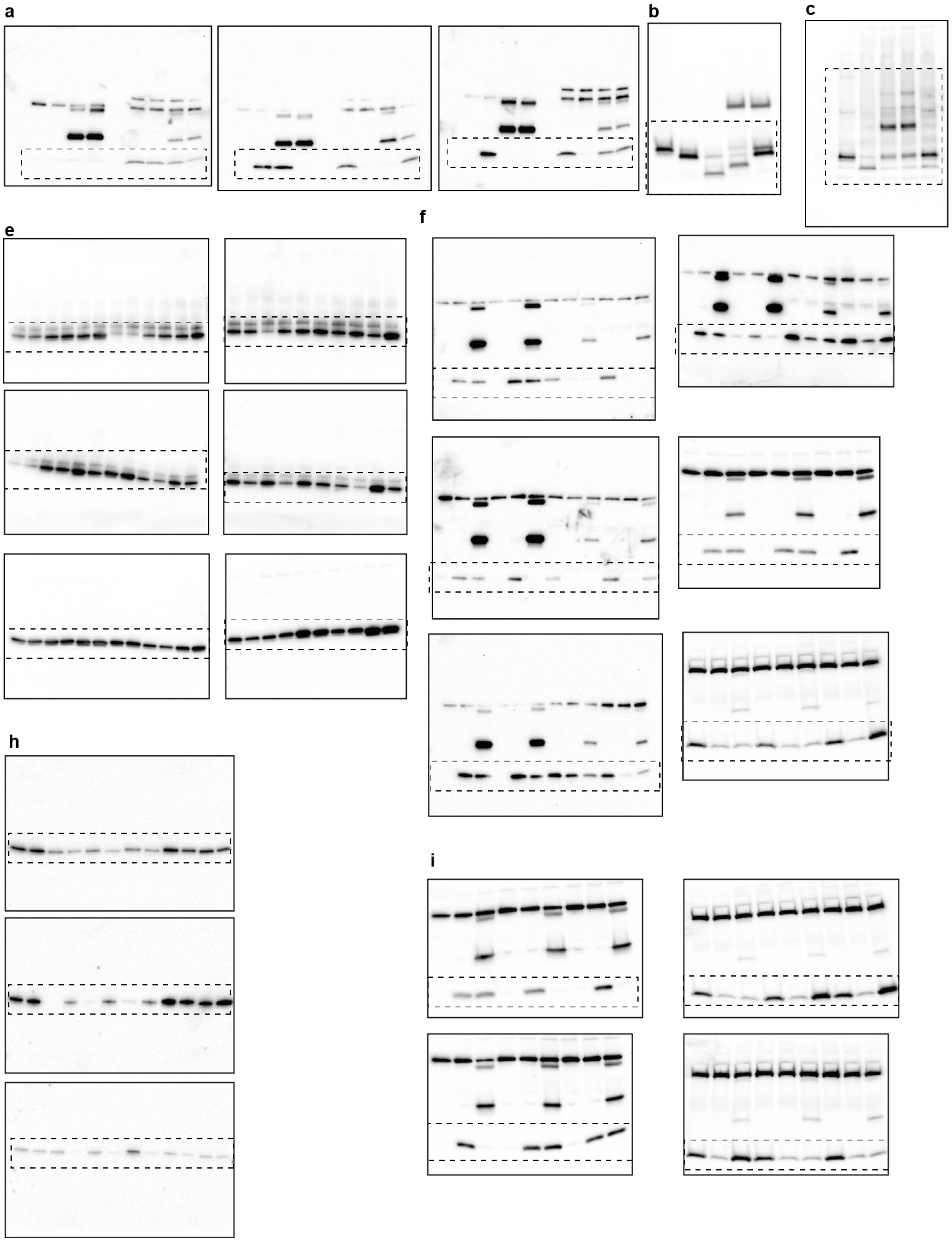

Supplementary Figure 11. Uncropped western blots from Figure 1.
The portion of each blot that appears in the Figure is indicated by a dotted outline.

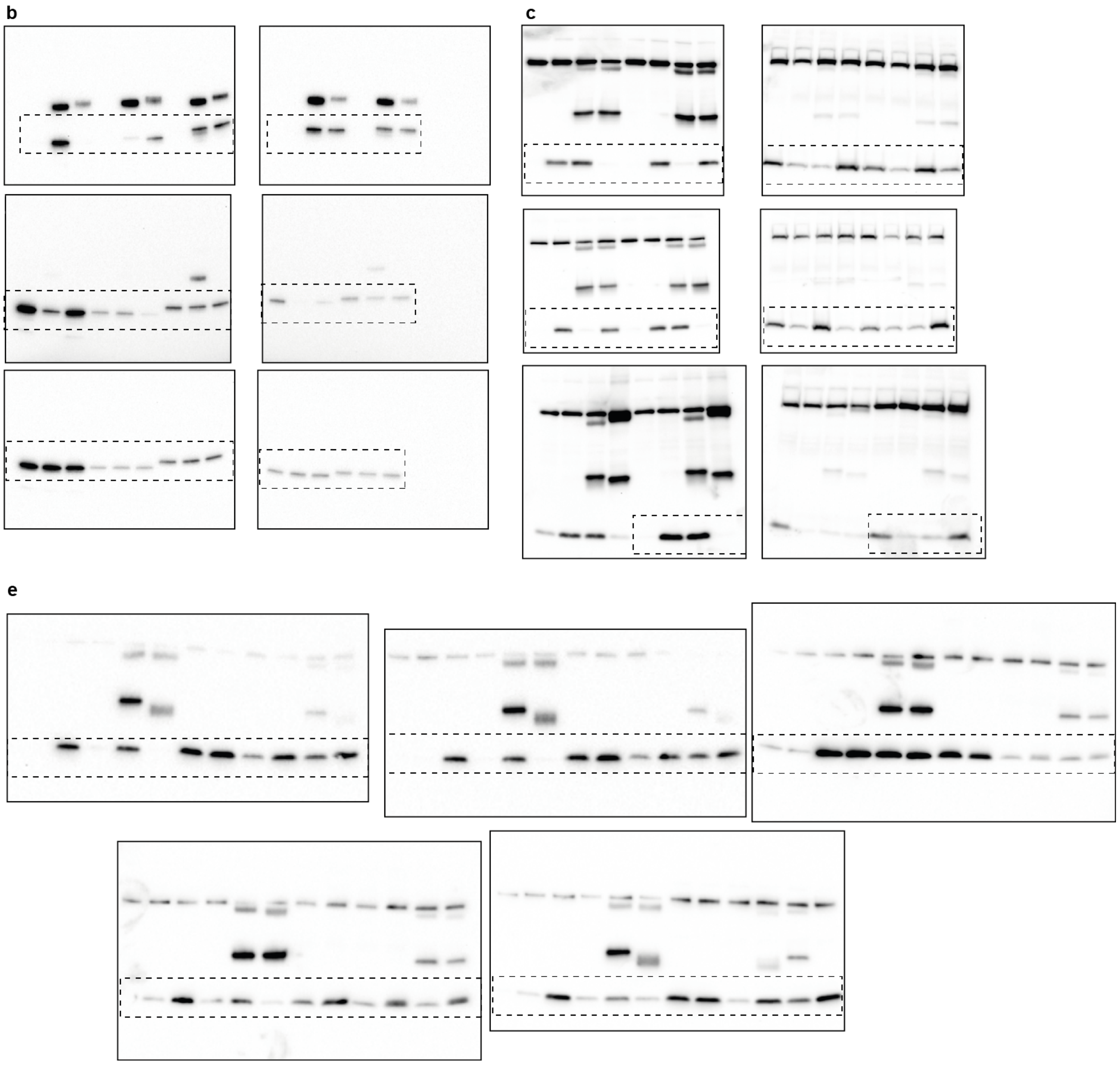

Supplementary Figure 12. Uncropped western blots from Figure 2.
The portion of each blot that appears in the Figure is indicated by a dotted outline.

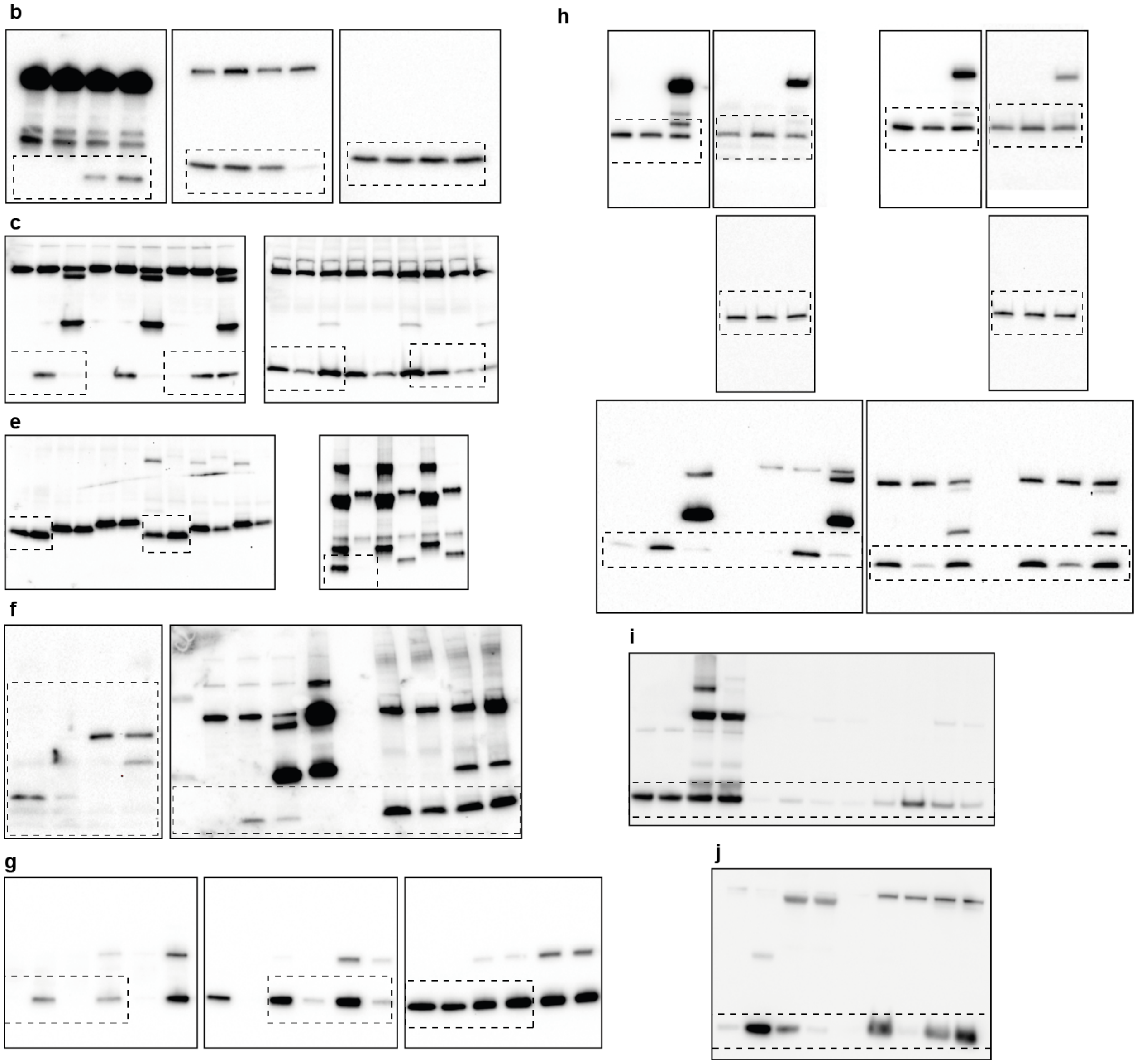

Supplementary Figure 13. Uncropped western blots from Figure 3.
The portion of each blot that appears in the Figure is indicated by a dotted outline.

Supplementary Table 1 – Primer sequences (5'-3') used for mutagenesis reactions

Primer	Primer 1 forward	Primer 2 reverse complement
hBak forward	tatatgaattccatggcttcggggcaaggccca	
hBak reverse	aagattcttcaaatcatgacgctcgagcggat	
hBak H43C	cgttttttaccgc**tgc**cagcaggaacag	ctgttcctgctggcagcggtaaaaaacg
hBak A49C	cagcaggaacaggagtgcgaaggggtggct	agccaccccttcgcactcctgttcctgctg
hBak E50C	gaacaggaggcttgcggggtggctgcc	ggcagccaccccgcaagcctcctgttc
hBak G51C	caggaggctgaatgcgtggctgcccctg	caggggcagccacgcattcagcctcctg
hBak V52C	ggaggctgaagggtgcgctgcccctgccg	cggcaggggcagcgcacccttcagcctcc
hBak A53C	ggctgaaggggtgtgcgcccctgccgacc	ggtcggcaggggcgcacaccccttcagcc
hBak P55C	aggggtggctgcctgcgccgacccagaga	tctctgggtcggcgcaggcagccacccct
hBak A56C	gggtggctgccccttgcgacccagagatggt	accatctctgggtcgcaaggggcagccaccc
hBak D57C	tggctgcccctgcctgcccagagatggtca	tgaccatctctgggcaggcaggggcagcca
hBak D57N	ggctgcccctgcc**aac**ccagagatggtca	tgaccatctctgggttggcaggggcagcc
hBak E59C	cctgccgacccatgcatggtcaccttac	gtaaggtgaccatgcatgggtcggcagg
hBak M60C	tgccgacccagagtgcgtcaccttacctct	agaggtaaggtgacgcactctgggtcggca
hBak T148C	ctgttcctgctggcagcggtaaaaaacg	tggcctaggaagccgcacaggccatgctgg
FLAG n-loop (replace)	caggaggctgaagactacaaggacgacgacgacaaggagatggc ac	gtgccatctccttgtcgtcgtcgtccttgtagtcttc agcctcctg
FLAG n-loop (insert)	aggctgaaggggggagactacaaggacgacgacgacaaggga- ggcgtggctgcccc	ggggcagccacgcctcccttgtcgtcgtcgtcct tgtagtctccccccttcagcct
FLAG m-loop	gccgacccagagggaggcgactacaaggacgacga- cgacaagggaggcatggtcacctta	taaggtgaccatgcctcccttgtcgtcgtcgtcctt gtagtcgcctccctctgggtcggc
FLAG c-loop	aacctagcagcggaggcgactacaaggacgacgacg- acaagggaggcaccatggggcag	ctgccccatggtgcctcccttgtcgtcgtcgtcctt gtagtcgcctccgctgctaggtt
mBak A50V	gagacccagggggtggccgcccctgcca	tggcaggggcggccaccccctgggtctc
mBak AEGVAAP	gcaggaacaggaggctgaaggggtggccgcccctgcca	tggcaggggcggccaccccttcagcctcctgtt cctgc
mBak AEGVAAPAD	gcaggaacaggaggccgagggggtggccgcccctgccgaccc- cgagatggacaac	gttgtccatctcggggtcggcaggggcggcca ccccctcggcctcctgttcctgc
mBak N55D	gcggccgcccctgccgaccccgagatggacaacttg	caagttgtccatctcggggtcggcaggggcgg ccgc
hBax forward	ggaattccatggacgggtccggggagc	
hBax GVAAP	ggggtggctgcccctgagctggccctggac	gtccagggccagctcaggggcagccacccc
hBax GVAAPAD	ccaggatcgagcaggggtggctgcccctgccgaccccgagct-ggc	gccagctcggggtcggcaggggcagccaccc ctgctcgatcctgg
